# Supplementary material for: Tropaeolin OO as a Chemical Sensor for a Trace Amount of Pd(II) Ions Determination
Source: Molecules. 2022 Jul 14;27(14):4511. doi: 10.3390/molecules27144511 (PMC9317805; doi:10.3390/molecules27144511)
Supplement: Supplementary file 1 [file molecules-27-04511-s001.zip › molecules-1749005-supplementary.pdf]

Supplementary Materials

Tropaeolin OO as a chemical sensor for trace amount of Pd(II) ions determination

Adrianna Pach[1], Agnieszka Podborska[2], Edit Csapo[3, 4] and Magdalena Luty-Błocho[1*]

[1] AGH University of Science and Technology, Faculty of Non-Ferrous Metals, A. Mickiewicza Av. 30, 30-059 Krakow, Poland.
[2] AGH University of Science and Technology, Academic Centre for Materials and Nanotechnology, A. Mickiewicza Av. 30, 30-059 Krakow, Poland
[3] MTA-SZTE Lendület "Momentum" Noble Metal Nanostructures Research Group, University of Szeged, H-6720 Rerrich B. sqr. 1, 6720 Szeged, Hungary
[4] Interdisciplinary Excellence Center, Department of Physical Chemistry and Materials Science, Universi-ty of Szeged, H-6720 Rerrich B. sqr. 1, 6720 Szeged, Hungary
* Correspondence: mlb@agh.edu.pl

1. Experimental conditions

Table S1. The conditions of experiments. The values of initial reagent concentration, i.e.: Pd(II) ions and tropaeolin OO before mixing. Total volume of reagents V = 4 mL.

Initial concentration of reagents		Volumetric ratio of TR to Pd(II) ions (sample notation)	T	pH
$C_{0,TR}$, mol/dm^3	$C_{0,Pd(II)}$, mol/dm^3	V_{TR} : $V_{Pd(II)}$, mL/mL	°C	
The stoichiometry				
		0.5 : 3.5 (A)	20	2.09 – 6.09
		1.0 : 3.0 (B)		(B – R)
$5\cdot 10^{-5}$	$5\cdot 10^{-5}$	1.5 : 2.5 (C)		
		2.0 : 2.0 (D)		and
		2.5 : 1.5 (E)		
		3.0 : 1.0 (F)		4 – 5
		3.5 : 0.5 (G)		(H_2O)
The influence of pH				
$5\cdot 10^{-5}$	$5\cdot 10^{-5}$	1.5 : 2.5	20	2.09
				2.87
				4.10
				5.02
				6.09
				7.00
The influence of temperature				
$5\cdot 10^{-5}$	$5\cdot 10^{-5}$	1.5 : 2.5	20	4.10
			30	
			40	
			50	
			60	
The influence of other anions (Cl^-, ClO_4^-) The concentration of anions in the sample was set as 0.075 mol/dm^3				
$5\cdot 10^{-5}$	$5\cdot 10^{-5}$	1.5 : 2.5	50	4.10
The influence of other cations (Na^+, K^+, Al^{3+}, Mg^{2+}, Co^{2+}, Ni^{2+}, Zn^{2+}, Pt^{2+})				

The concentration of cations in the sample was set as 0.075 mol/dm^3				
5· 10^{-5}	5· 10^{-5}	1.5 : 2.5	50	4.10
The Pd(II) quantitative determination in the presence of Pt(IV)				
5· 10^{-5}	5· 10^{-5}	1.5 : 2.5	60	4.10
5· 10^{-6}	5· 10^{-6}			
1· 10^{-5}	1· 10^{-5}			
1· 10^{-4}	1· 10^{-4}			
2· 10^{-4}	2· 10^{-4}			

2. Spectra of reagents

Before synthesizing metalorganic complexes, the proper reagents i.e. solution of tropaeolin OO (TR) and Pd(II) ions in H_2O and B-R buffer (pH = 2.09 – 6.09) as solvent were spectrophotometrically analyzed. Obtained spectra for an aqueous solution of TR and determining molar coefficient (ε) were shown in Fig. S1 – Fig. S6.

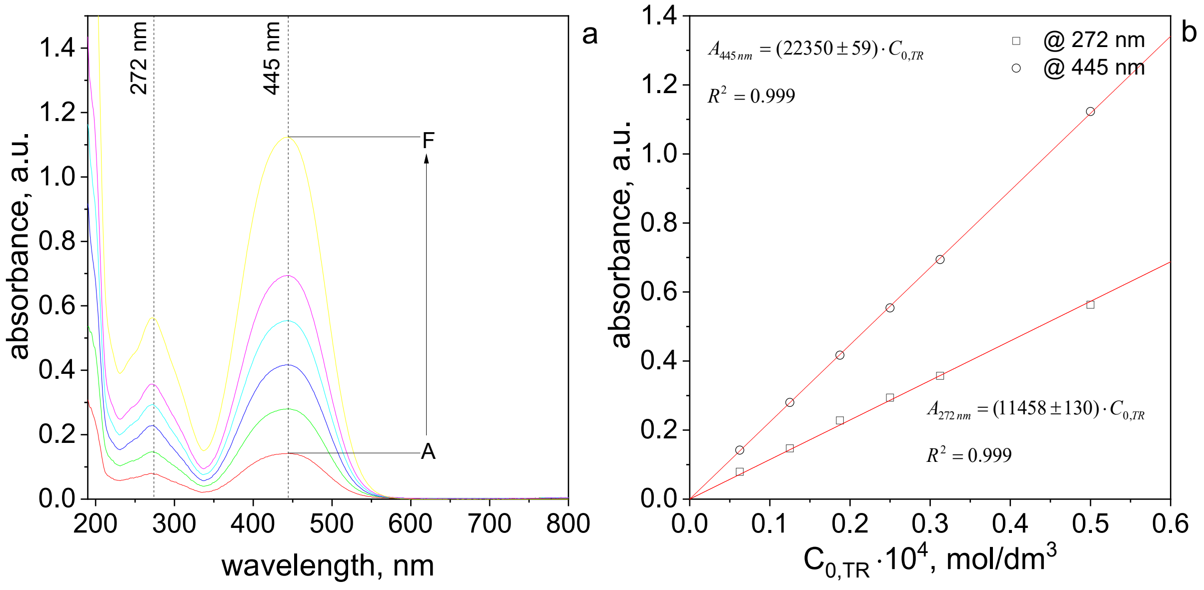

(**a**) (**b**)

Figure S1. The spectra UV-Vis of tropaeolin OO aqueous solutions (H_2O as solvent) with different initial concentrations: A – 6.25·10^{-6} mol/dm^3; B – 1.25·10^{-5} mol/dm^3; C – 1.87·10^{-5} mol/dm^3; D – 2.5·10^{-5} mol/dm^3; E – 3.125·10^{-5} mol/dm^3; F – 5·10^{-5} mol/dm^3 (a), dependency of absorbance vs. initial concentrations of tropaeolin OO in the range 6.25·10^{-6} mol/dm^3 to 5·10^{-5} mol/dm^3, wavelength: 272 nm and 445 nm, (b). Conditions: T = 20°C, path length 1 cm.

The aqueous solutions of TR have a characteristic UV-Vis spectrum with two maximum localized at 272 nm and 445 nm (Fig. S1a). According to Lambert-Beer law, the intensity of the spectrum is directly proportional to the concentration of dye (Fig. S1b). Based on these results (absorbance vs. concentration, Fig. S1b), the values of molar coefficients from the slope of the fitted curve to experimental data were determined and equal ε (272 nm) = 11 458 ± 130 $M^{-1}\cdot cm^{-1}$ and ε (445 nm) = 22 350 ± 59 $M^{-1}\cdot cm^{-1}$.

Analogous spectra were registered for TR in the B-R buffer solution. The obtained spectra and graphically determining values of molar coefficient at different pH of the solution were shown in Fig. S2 – S6.

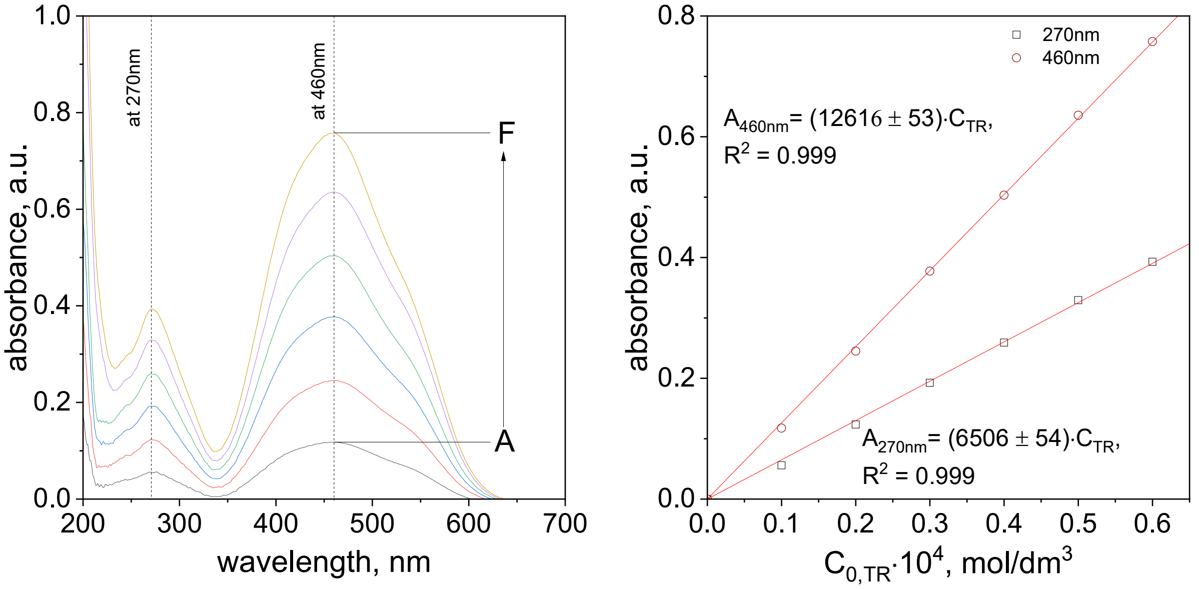

(**a**) (**b**)

Figure S2. The spectra UV-Vis of tropaeolin OO aqueous solutions (B – R buffer as solvent) at different initial concentrations: A – $6.25\cdot 10^{-6}$ mol/dm^3; B – $1.25\cdot 10^{-5}$ mol/dm^3; C – $1.87\cdot 10^{-5}$ mol/dm^3; D – $2.5\cdot 10^{-5}$ mol/dm^3; E – $3.125\cdot 10^{-5}$ mol/dm^3; F – $5\cdot 10^{-5}$ mol/dm^3 (a), dependency of absorbance vs. initial concentrations of tropaeolin OO in the range $6.25\cdot 10^{-6}$ mol/dm^3 to $5\cdot 10^{-5}$ mol/dm^3, wavelength: 270 nm and 460 nm, (b). Conditions: T = 20°C, path length 1 cm, pH= 2.09.

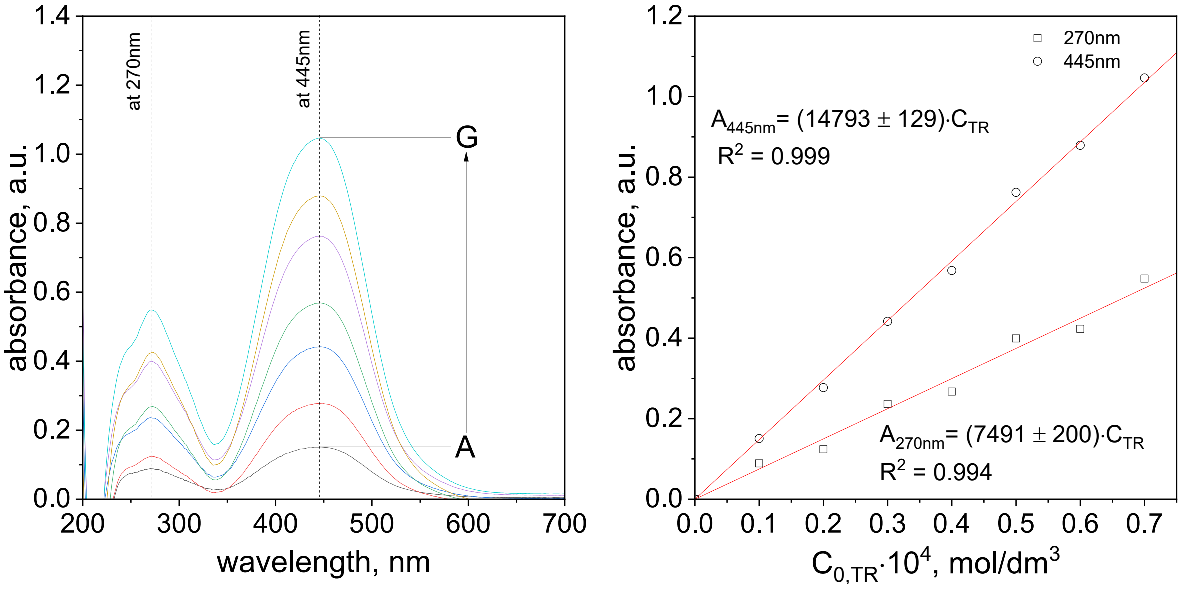

(**a**) (**b**)

Figure S3. The spectra UV-Vis of tropaeolin OO aqueous solutions (B – R buffer as solvent) at different initial concentrations: A – 6.25·10^{-6} mol/dm^3; B – 1.25·10^{-5} mol/dm^3; C – 1.87·10^{-5} mol/dm^3; D – 2.5·10^{-5} mol/dm^3; E – 3.125·10^{-5} mol/dm^3; F – 5·10^{-5} mol/dm^3 (a), dependency of absorbance vs. initial concentrations of tropaeolin OO in the range 6.25·10^{-6} mol/dm^3 to 5·10^{-5} mol/dm^3, wavelength: 270 nm and 445 nm, (b). Conditions: T = 20°C, path length 1 cm, pH= 2.87

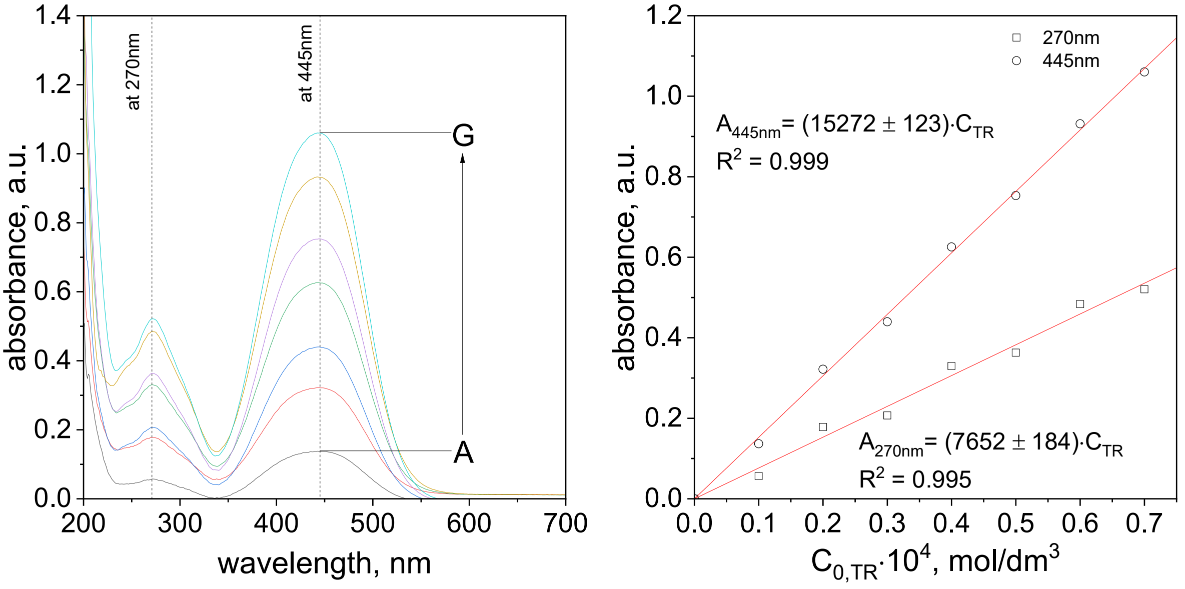

(**a**) (**b**)

Figure S4. The spectra UV-Vis of tropaeolin OO aqueous solutions (B – R buffer as solvent) at different initial concentrations: A – 6.25·10^{-6} mol/dm^3; B – 1.25·10^{-5} mol/dm^3; C – 1.87·10^{-5} mol/dm^3; D – 2.5·10^{-5} mol/dm^3; E – 3.125·10^{-5} mol/dm^3; F – 5·10^{-5} mol/dm^3 (a), dependency of absorbance vs. initial concentrations of tropaeolin OO in the range 6.25·10^{-6} mol/dm^3 to 5·10^{-5} mol/dm^3, wavelength: 270 nm and 460 nm, (b). Conditions: T = 20°C, path length 1 cm, pH= 4.10

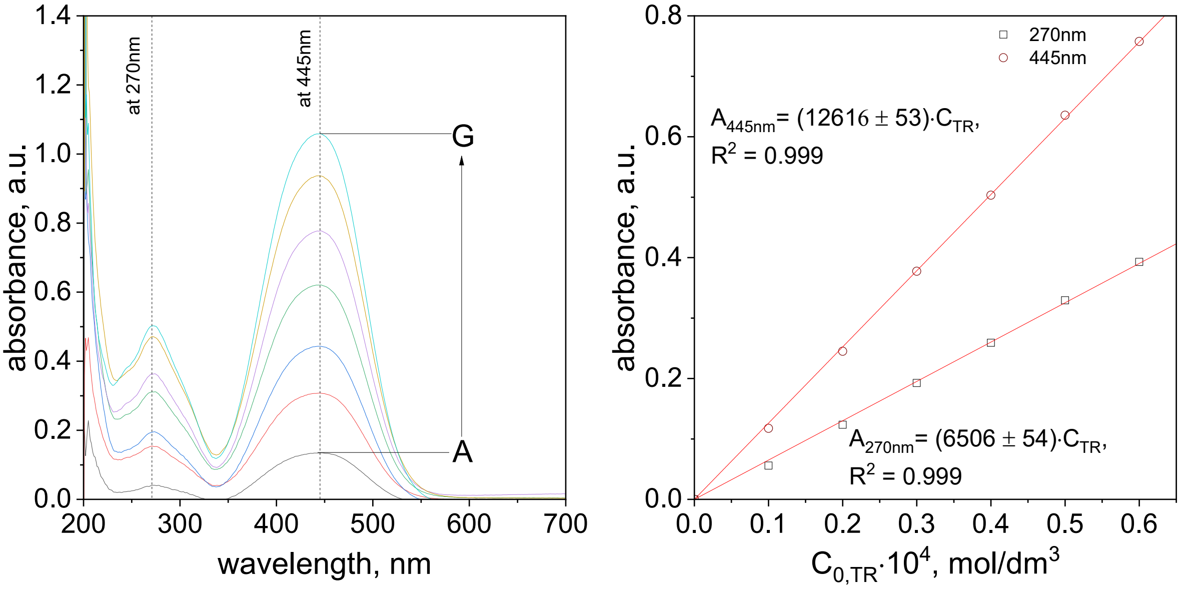

(**a**) (**b**)

Figure S5. The spectra UV-Vis of tropaeolin OO aqueous solutions (B – R buffer as solvent) at different initial concentrations: A – 6.25·10^{-6} mol/dm^3; B – 1.25·10^{-5} mol/dm^3; C – 1.87·10^{-5} mol/dm^3; D – 2.5·10^{-5} mol/dm^3; E – 3.125·10^{-5} mol/dm^3; F – 5·10^{-5} mol/dm^3 (a), dependency of absorbance vs. initial concentrations of tropaeolin OO in the range 6.25·10^{-6} mol/dm^3 to 5·10^{-5} mol/dm^3, wavelength: 270 nm and 445 nm, (b). Conditions:, T = 20°C, path length 1 cm, pH= 5.02.

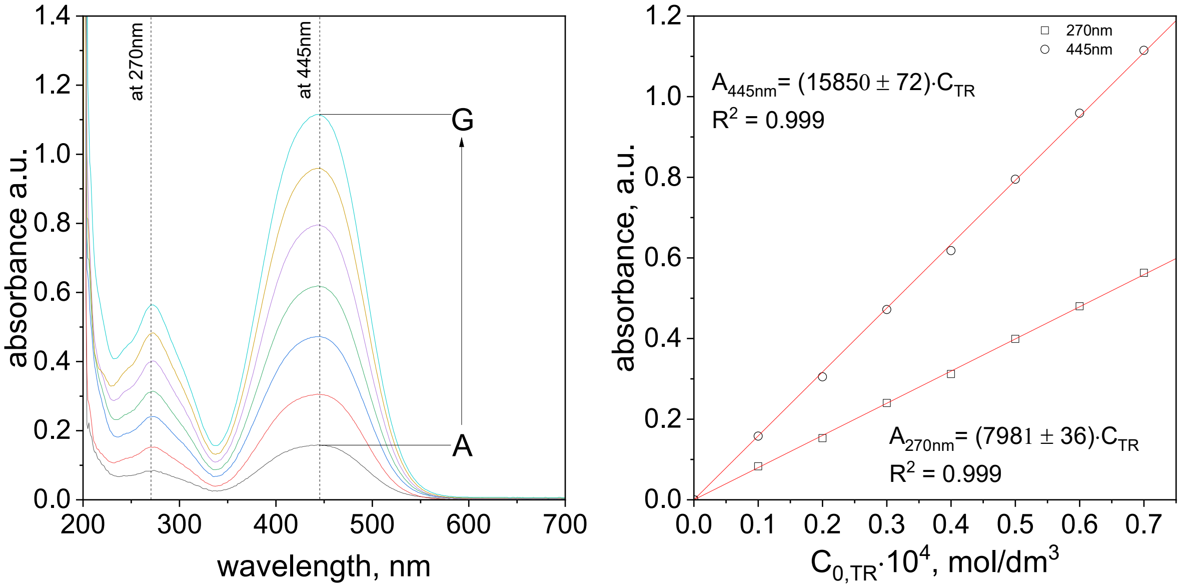

(**a**) (**b**)

Figure S6. The spectra UV-Vis of tropaeolin OO aqueous solutions (B – R buffer as solvent) at different initial concentrations: A – 6.25·10^{-6} mol/dm^3; B – 1.25·10^{-5} mol/dm^3; C – 1.87·10^{-5} mol/dm^3; D – 2.5·10^{-5} mol/dm^3; E – 3.125·10^{-5} mol/dm^3; F – 5·10^{-5} mol/dm^3 (a), dependency of absorbance vs. initial concentrations of tropaeolin OO in the range 6.25·10^{-6} mol/dm^3 to 5·10^{-5} mol/dm^3, wavelength: 270 nm and 445 nm, (b). Conditions:, T = 20°C, path length 1 cm, pH= 6.09

The characteristic UV-Vis spectra and graphical determined value of molar coefficient were registered for Pd(II) aqueous solution and shown in Fig. S7.

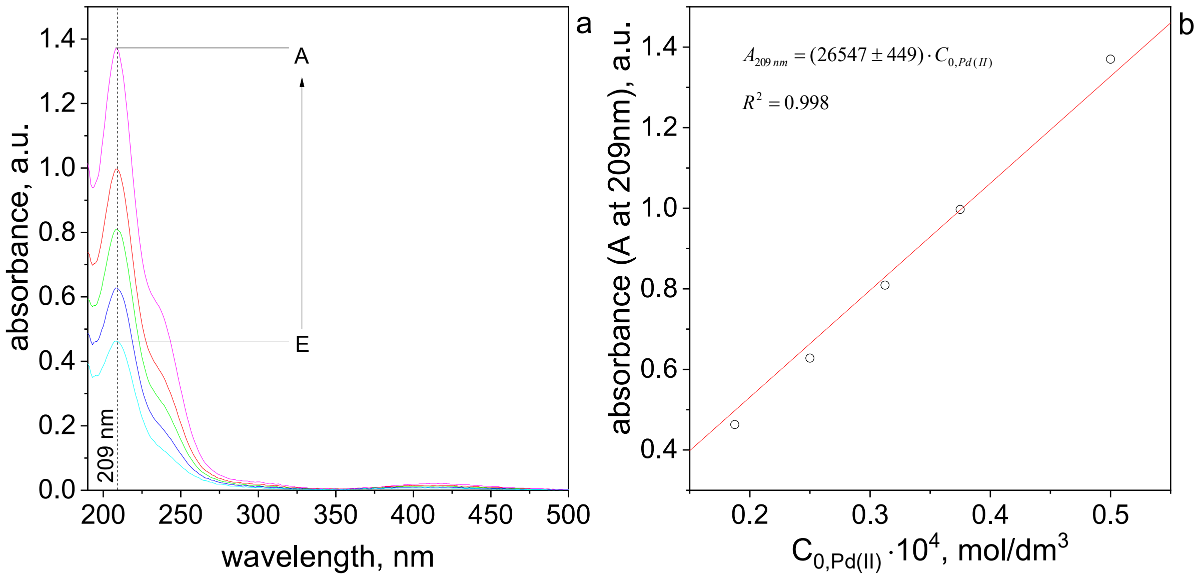

(**a**) (**b**)

Figure S7. The spectra UV-Vis of Pd(II) aqueous solutions (H_2O as solvent) with different initial concentrations: A – $5 \cdot 10^{-5}$ M; B – $3.75 \cdot 10^{-5}$ M; C – $3.125 \cdot 10^{-5}$ M; D – $2.5 \cdot 10^{-5}$ M; E – $1.875 \cdot 10^{-5}$ M (a), dependency of absorbance vs. initial concentrations of Pd(II) in the range $1.875 \cdot 10^{-5}$ M to $5 \cdot 10^{-5}$ M (b). Conditions: T = 20°C, path length 1 cm.

The aqueous solutions of Pd(II) ions have a characteristic UV-Vis spectrum with two maximum and two shoulders (between 220 and 320 nm) (Fig. S7a). One strong peak is located at 209 nm whereas the second with small intensity at around 420 nm. Based on obtained spectra, the values of the molar coefficient were determined from the slope of the fitted curve to experimental data (Fig. S7b) and it equals ε (209 nm) = 26 547 ± 449 $M^{-1} \cdot cm^{-1}$. Registered UV-Vis spectra (Fig. S1a and S7a) of reagents show, that in the range of wavelength 200 – 350 nm they overlap.

3. Metalorganic complex formation between Pd(II) and TR (at different pH)

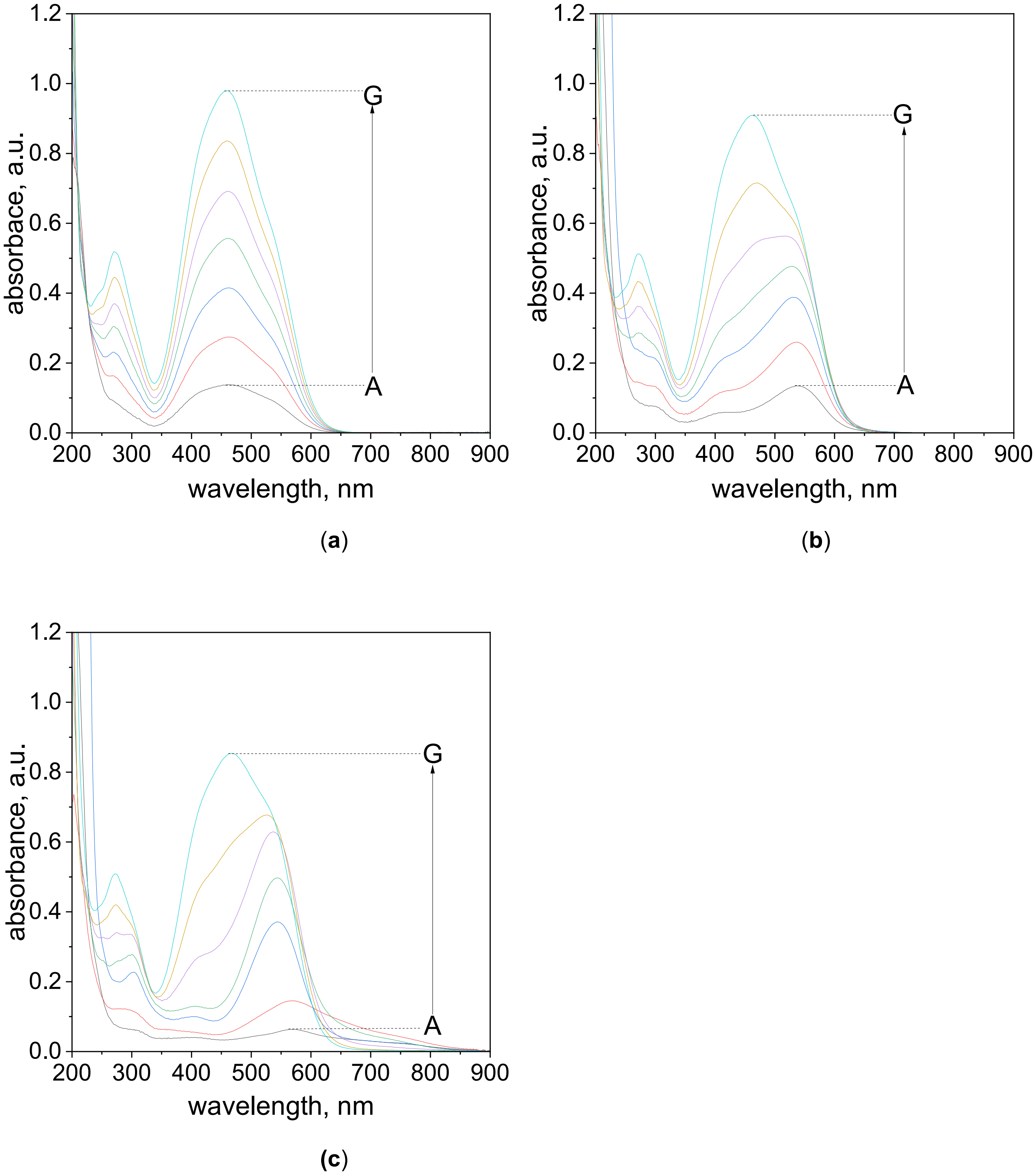

Figure S8. The UV-Vis spectra of solutions obtained after mixing of Pd(**II**) ions with tropaeolin at different volumetric ratio ($V_{Pd(II)}$: V_{TR}): 3.5 : 0.5 (A); 3.0 : 1.0 (B); 2.5 : 1.5 (C); 2.0 : 2.0 (D), 1.5 : 2.5 (E); 1.0 : 3.0 (F), 0.5 : 3.5 (G) after: 5 min (a), 24 hours (b), 7days (c). Conditions: $C_{0,\ Pd(II)}$ = $C_{0,\ TR}$ = $5 \cdot 10^{-5}$ mol/dm^3 (before reagent mixing), T = 20°C, pH = 2.09.

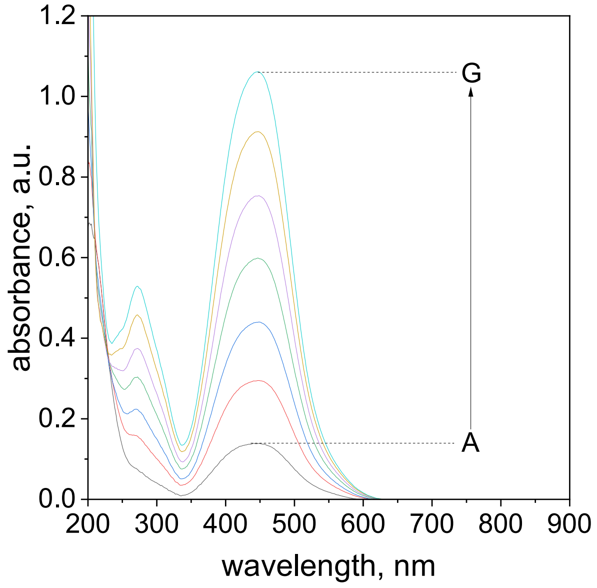

(**a**)

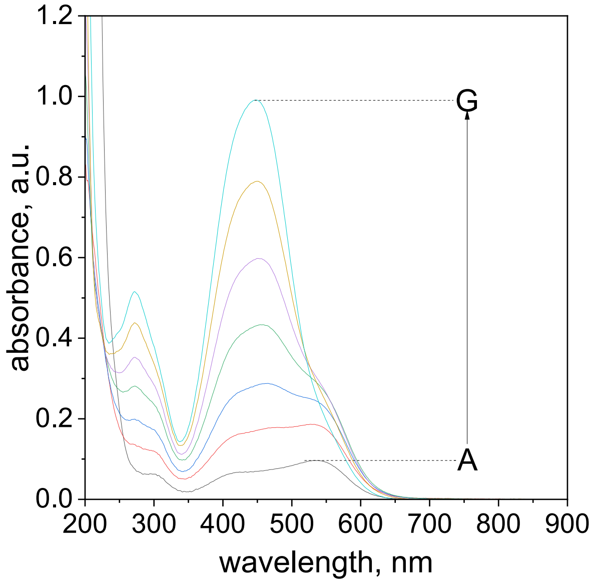

(**b**)

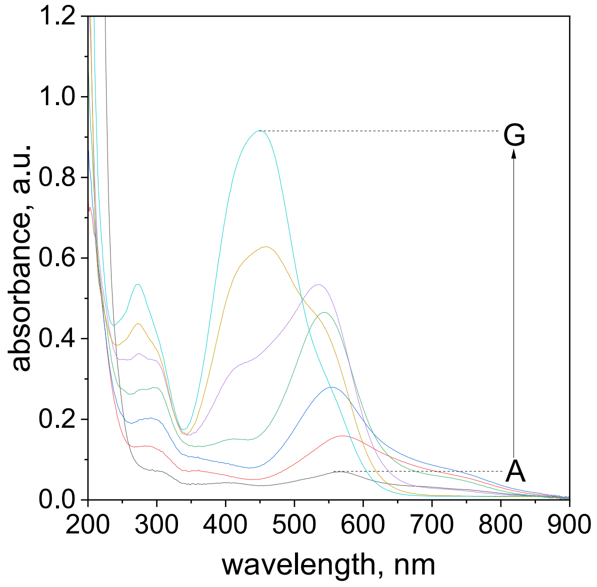

(c)

Figure S9. The UV-Vis spectra of solutions obtained after mixing of Pd(**II**) ions with tropaeolin at different volumetric ratio ($V_{Pd(II)}$: V_{TR}) 3.5 : 0.5 (A); 3.0 : 1.0 (B); 2.5 : 1.5 (C); 2.0 : 2.0 (D), 1.5 : 2.5 (E); 1.0 : 3.0 (F), 0.5 : 3.5 (G) after: 5 min (a), 24 hours (b), 7days (c). Conditions: $C_{0,\ Pd(II)}$ = $C_{0,\ TR}$ = $5{\cdot}10^{-5}$ mol/dm^3 (before reagent mixing), T = 20°C, pH = 2.*87*.

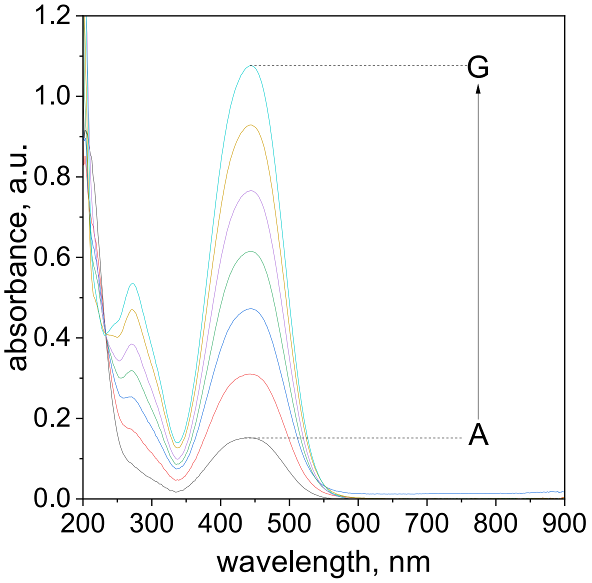

(**a**)

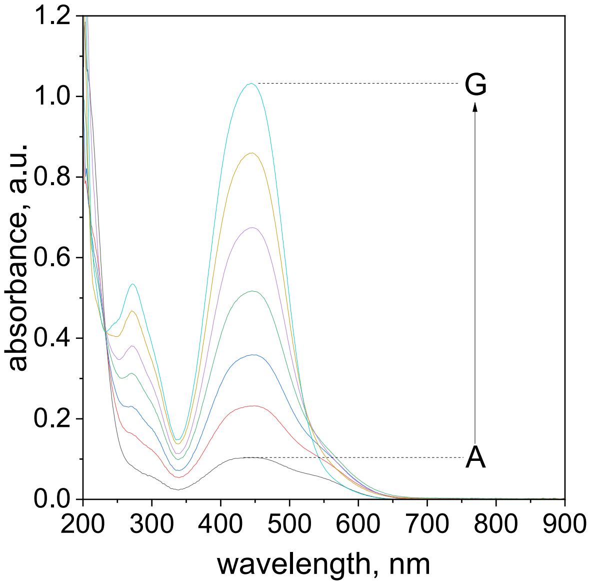

(**b**)

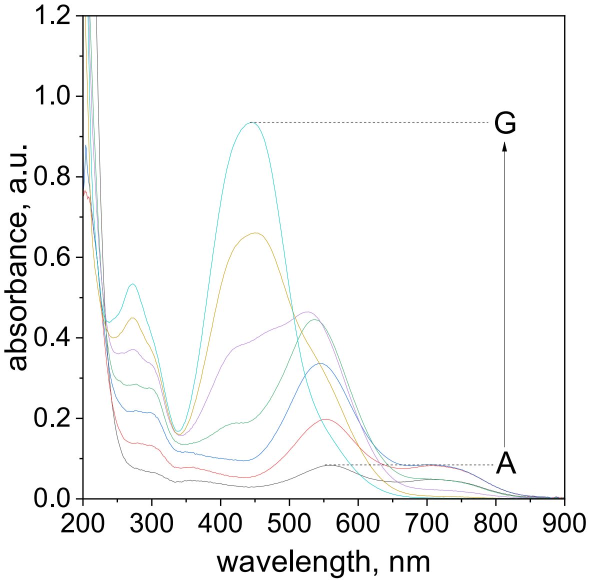

(c)

Figure S10. The UV-Vis spectra of solutions obtained after mixing of Pd(**II**) ions with tropaeolin at different volumetric ratio ($V_{Pd(II)}$: V_{TR}) 3.5 : 0.5 (A); 3.0 : 1.0 (B); 2.5 : 1.5 (C); 2.0 : 2.0 (D), 1.5 : 2.5 (E); 1.0 : 3.0 (F), 0.5 : 3.5 (G) after: 5 min (a), 24 hours (b), 7days (c). Conditions: $C_{0,\ Pd(II)}$ = $C_{0,\ TR}$ = $5{\cdot}10^{-5}$ mol/dm^3 (before reagent mixing), T = 20°C, pH = 4.10.

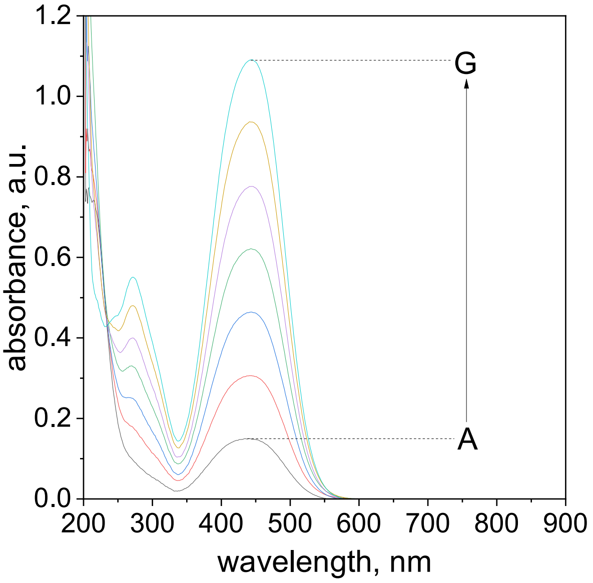

(**a**)

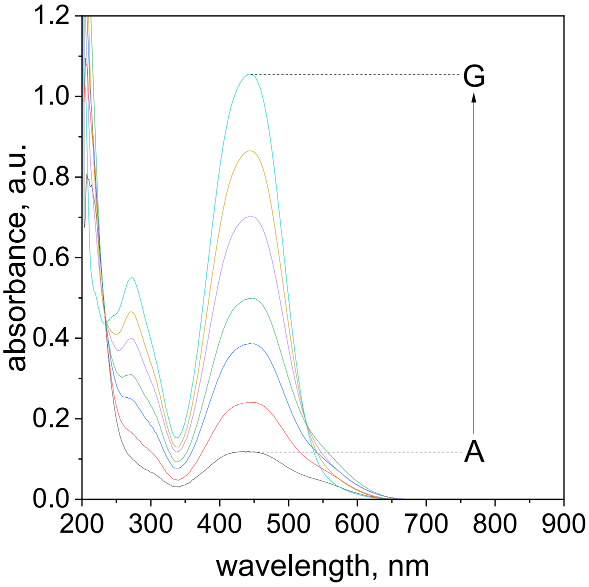

(**b**)

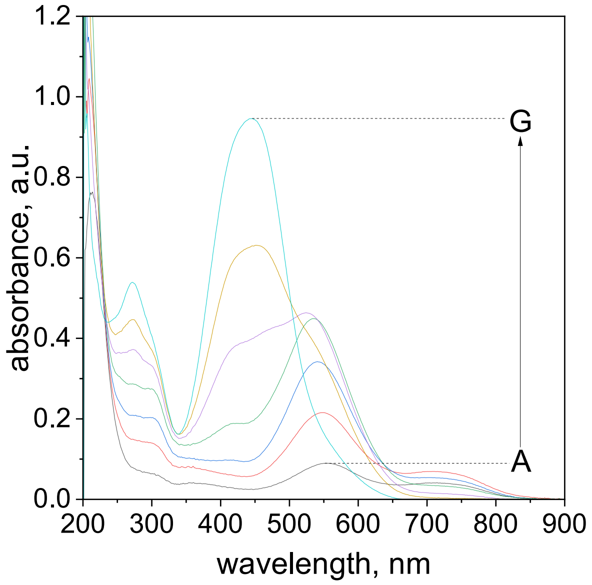

(c)

Figure S11. The UV-Vis spectra of solutions obtained after mixing of Pd(**II**) ions with tropaeolin at different volumetric ratio ($V_{Pd(II)}$: V_{TR}) 3.5 : 0.5 (A); 3.0 : 1.0 (B); 2.5 : 1.5 (C); 2.0 : 2.0 (D), 1.5 : 2.5 (E); 1.0 : 3.0 (F), 0.5 : 3.5 (G) after*:* 5 min (a), 24 hours (b), 7days (c). Conditions: $C_{0,\ Pd(II)}$ = $C_{0,\ TR}$ = $5\cdot10^{-5}$ mol/dm^3 (before reagent mixing), T = 20°C, pH = 5.02

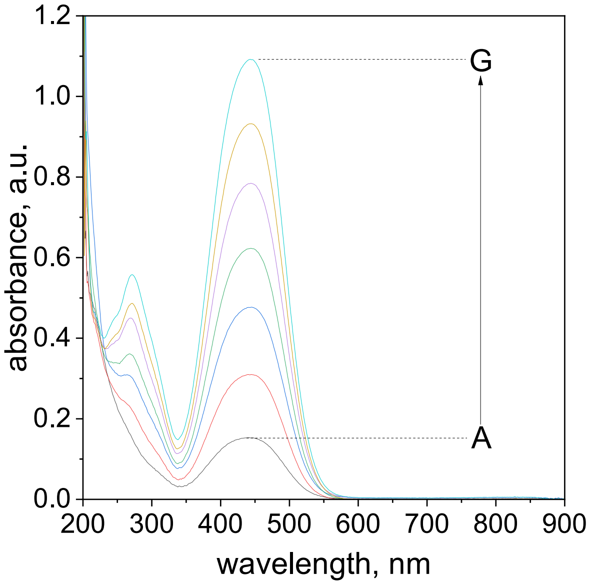

(**a**)

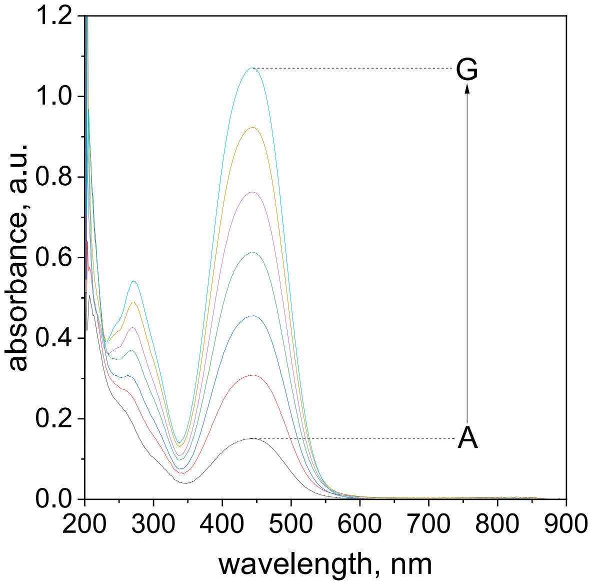

(**b**)

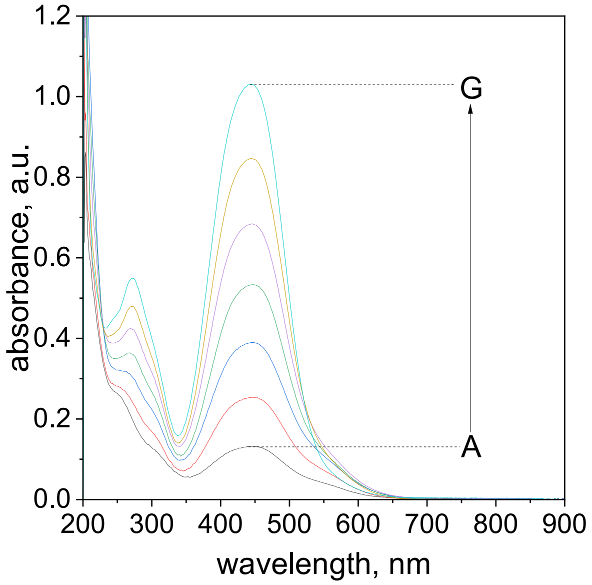

(c)

Figure S12. The UV-Vis spectra of solutions obtained after mixing of Pd(**II**) ions with tropaeolin at different volumetric ratio ($V_{Pd(II)}$: V_{TR}) 3.5 : 0.5 (A); 3.0 : 1.0 (B); 2.5 : 1.5 (C); 2.0 : 2.0 (D), 1.5 : 2.5 (E); 1.0 : 3.0 (F), 0.5 : 3.5 (G) after*:* 5 min (a), 24 hours (b), 7days (c). Conditions: $C_{0, Pd(II)}$ = $C_{0, TR}$ = $5 \cdot 10^{-5}$ mol/dm^3 (before reagent mixing), T = 20°C, pH = 6.09.

Job's plot

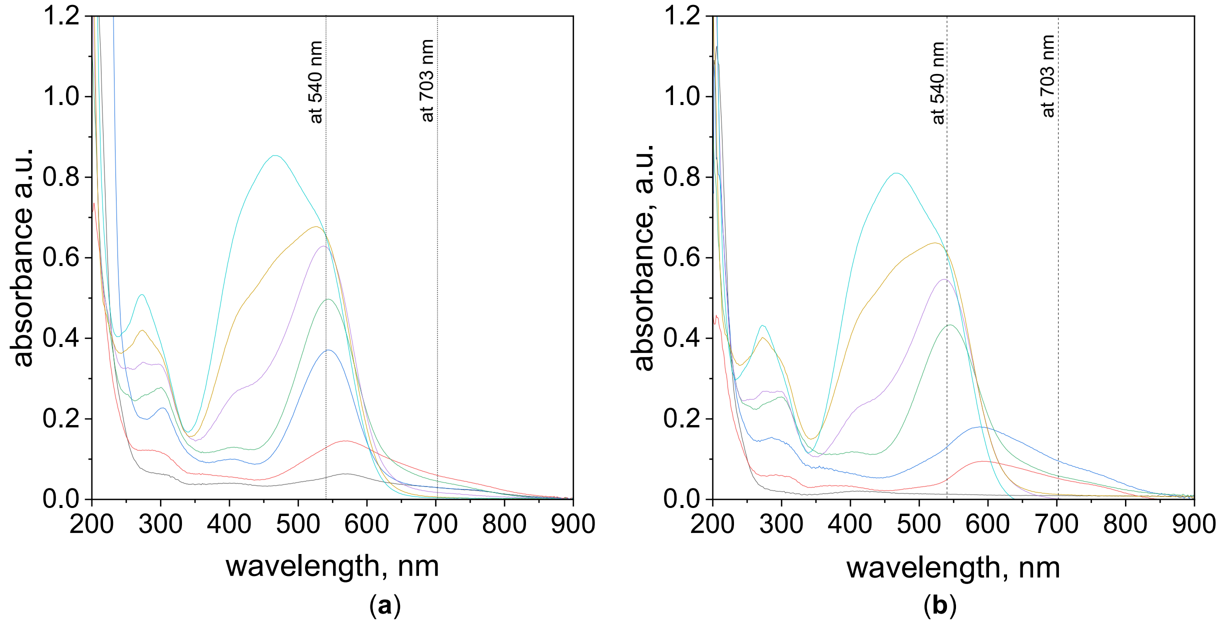

Figure S13. The UV-Vis spectra of solutions obtained after mixing of Pd(II) ions with tropaeolin at different volumetric ratio ($V_{Pd(II)}$: V_{TR}) 3.5 : 0.5 (A); 3.0 : 1.0 (B); 2.5 : 1.5 (C); 2.0 : 2.0 (D), 1.5 : 2.5 (E); 1.0 : 3.0 (F), 0.5 : 3.5 (G), past 24h (a) and 7 days (b). Conditions: $C_{0,TR} = C_{0,Pd(II)} = 5 \cdot 10^{-5}$ mol/dm^3 (before reagent mixing), T = 20°C, pH = 2.09.

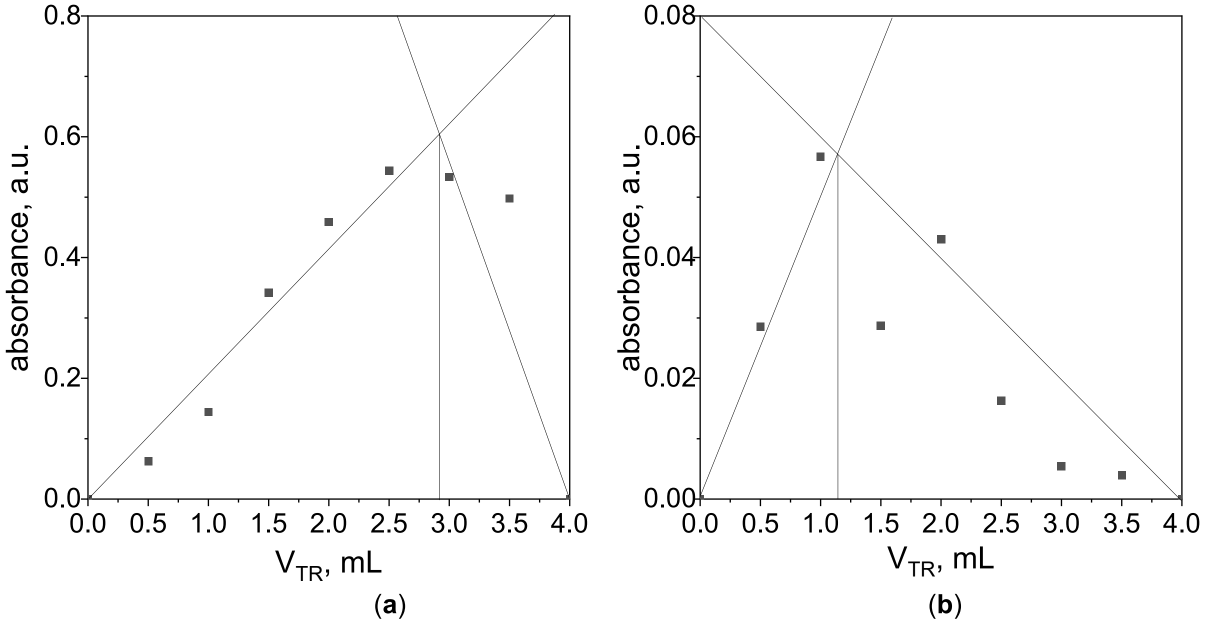

Figure S14. The dependency of absorbance vs. ratio of tropaeolin to Pd(II) volumes, registered at 540 nm (a); and at 703 nm (b) past 24h. Conditions: $C_{0,TR} = C_{0,Pd(II)} = 5 \cdot 10^{-5}$ mol/dm^3, total volume of reagent = 4.0 mL, T = 20°C, pH = 2.09.

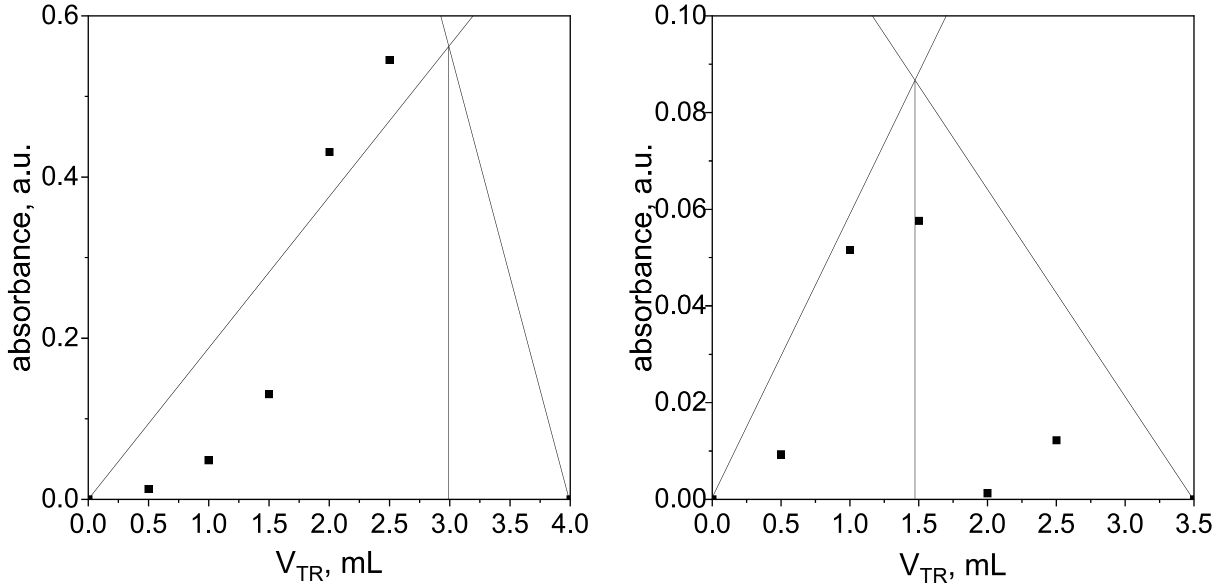

(**a**) (**b**)

Figure S15. The dependency of absorbance vs. ratio of tropaeolin to Pd(II) volumes, registered at 540 nm (a); and at 703 nm (b) past 7 days. Conditions: $C_{0,TR} = C_{0,Pd(II)} = 5\cdot10^{-5}$ mol/dm^3, total volume of reagent = 4.0 mL, T = 20°C, pH = 2.09.

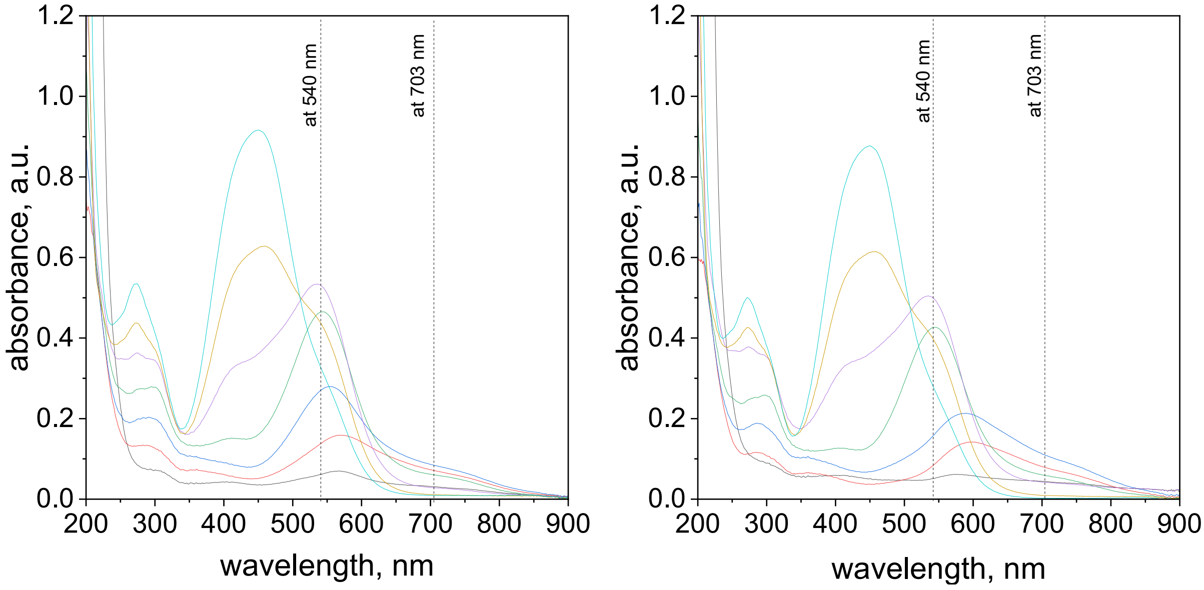

(**a**) (**b**)

Figure S16. The UV-Vis spectra of solutions obtained after mixing of Pd(II) ions with tropaeolin at different volumetric ratio ($V_{Pd(II)} : V_{TR}$) 3.5 : 0.5 (A); 3.0 : 1.0 (B); 2.5 : 1.5 (C); 2.0 : 2.0 (D), 1.5 : 2.5 (E); 1.0 : 3.0 (F), 0.5 : 3.5 (G), past 24h (a) and 7 days (b). Conditions: $C_{0,TR} = C_{0,Pd(II)} = 5\cdot10^{-5}$ mol/dm^3 (before reagent mixing), T = 20°C, pH = 2.87.

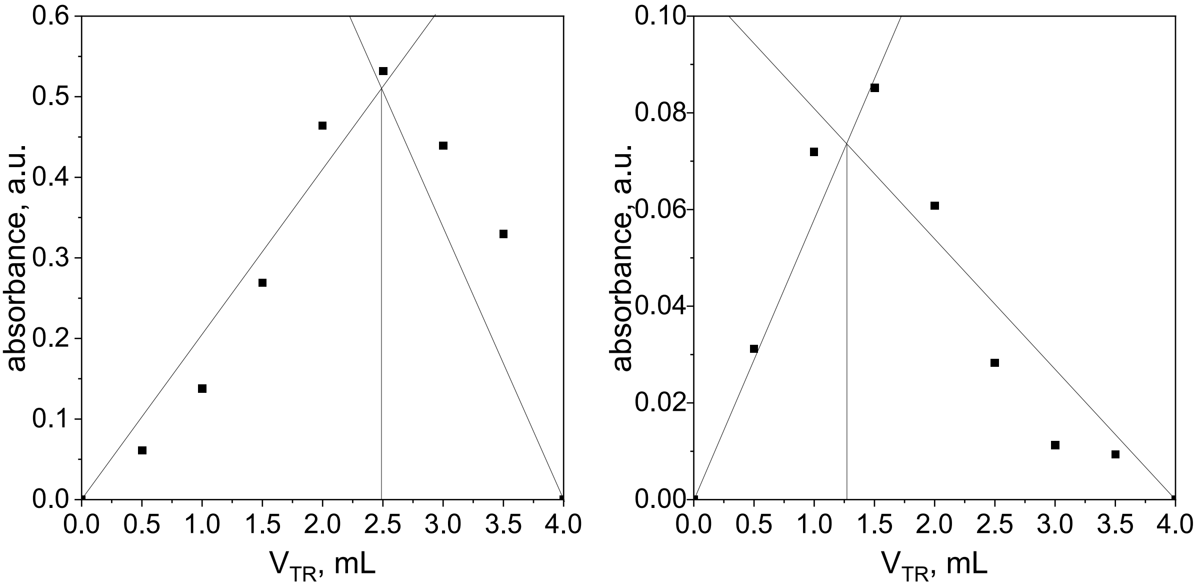

(**a**) (**b**)

Figure S17. The dependency of absorbance vs. ratio of tropaeolin to Pd(II) volumes, registered at 540 nm (b); and at 703 nm (c) past 24h. Conditions: $C_{0,TR} = C_{0,Pd(II)} = 5 \cdot 10^{-5}$ mol/dm^3, total volume of reagent = 4.0 mL, T = 20°C, pH = 2.87.

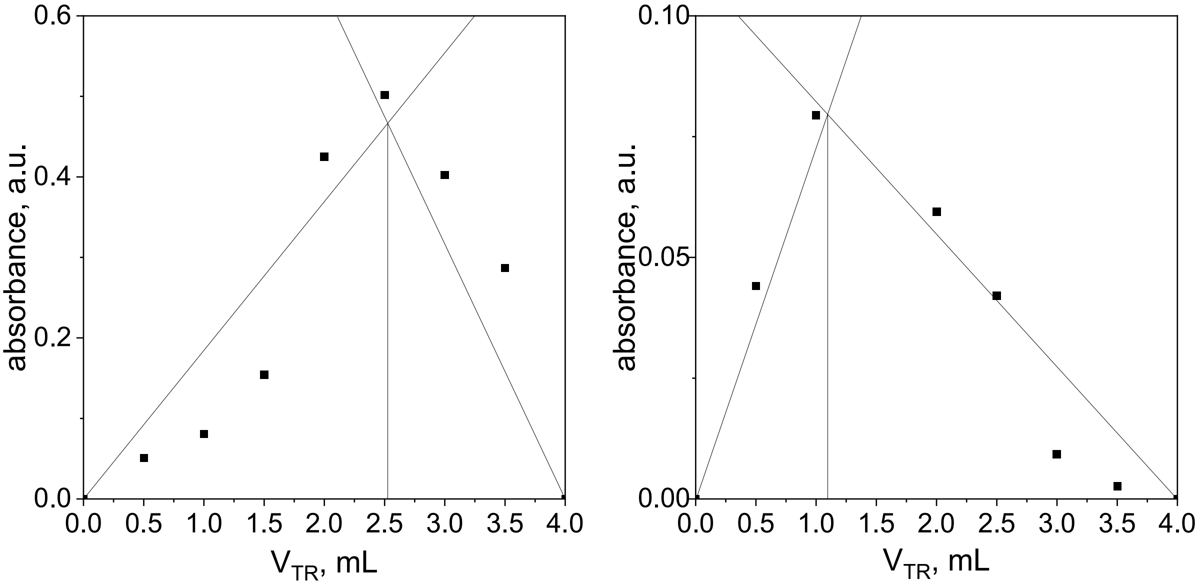

(**a**) (**b**)

Figure S18. The dependency of absorbance vs. ratio of tropaeolin to Pd(II) volumes, registered at 540 nm (a); and at 703 nm (b) past 7 days. Conditions: $C_{0,TR} = C_{0,Pd(II)} = 5 \cdot 10^{-5}$ mol/dm^3, total volume of reagent = 4.0 mL, T = 20°C, pH = 2.87.

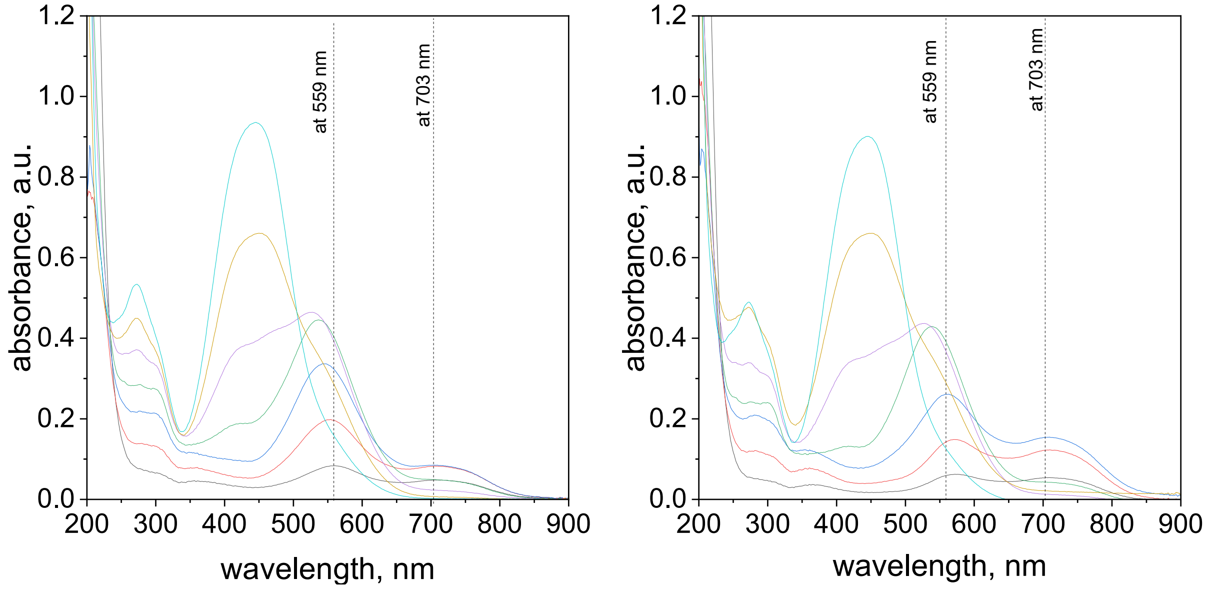

(**a**) (**b**)

Figure S19. The UV-Vis spectra of solutions obtained after mixing of Pd(II) ions with tropaeolin at different volumetric ratio ($V_{Pd(II)}$: V_{TR}) 3.5 : 0.5 (A); 3.0 : 1.0 (B); 2.5 : 1.5 (C); 2.0 : 2.0 (D), 1.5 : 2.5 (E); 1.0 : 3.0 (F), 0.5 : 3.5 (G), past 24h (a) and 7 days (b). Conditions: $C_{0,TR} = C_{0,Pd(II)} = 5{\cdot}10^{-5}$ mol/dm^3 (before reagent mixing), T = 20°C, pH = 4.10.

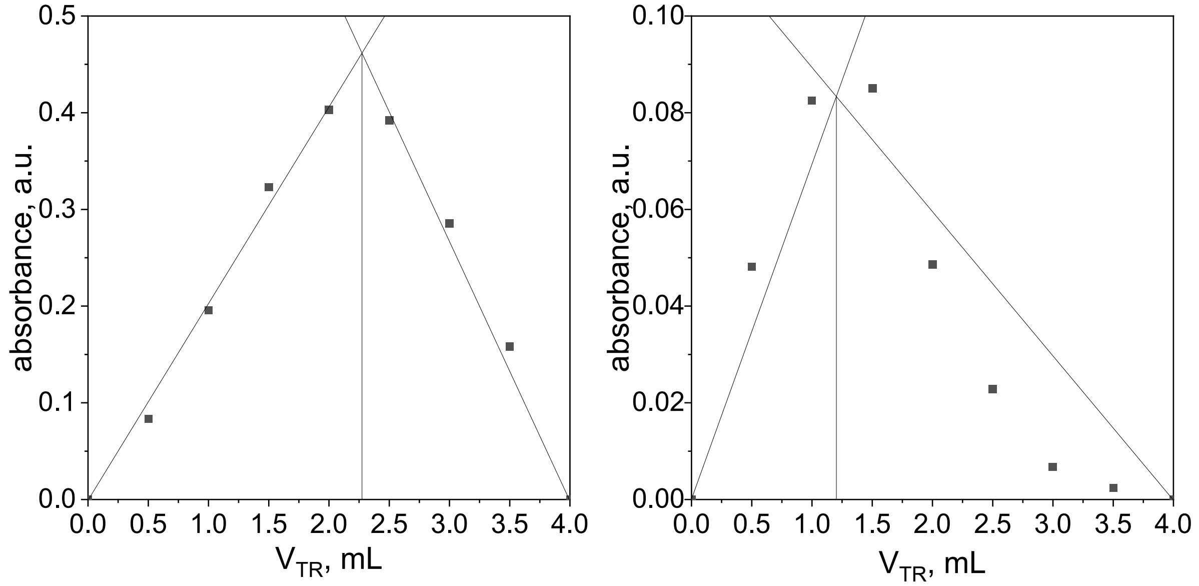

(**a**) (**b**)

Figure S20. The dependency of absorbance vs. ratio of tropaeolin to Pd(II) volumes, registered at 559 nm (b); and at 703 nm (c) past 24h. Conditions: $C_{0,TR} = C_{0,Pd(II)} = 5{\cdot}10^{-5}$ mol/dm^3, total volume of reagent = 4.0 mL, T = 20°C, pH = 4.10.

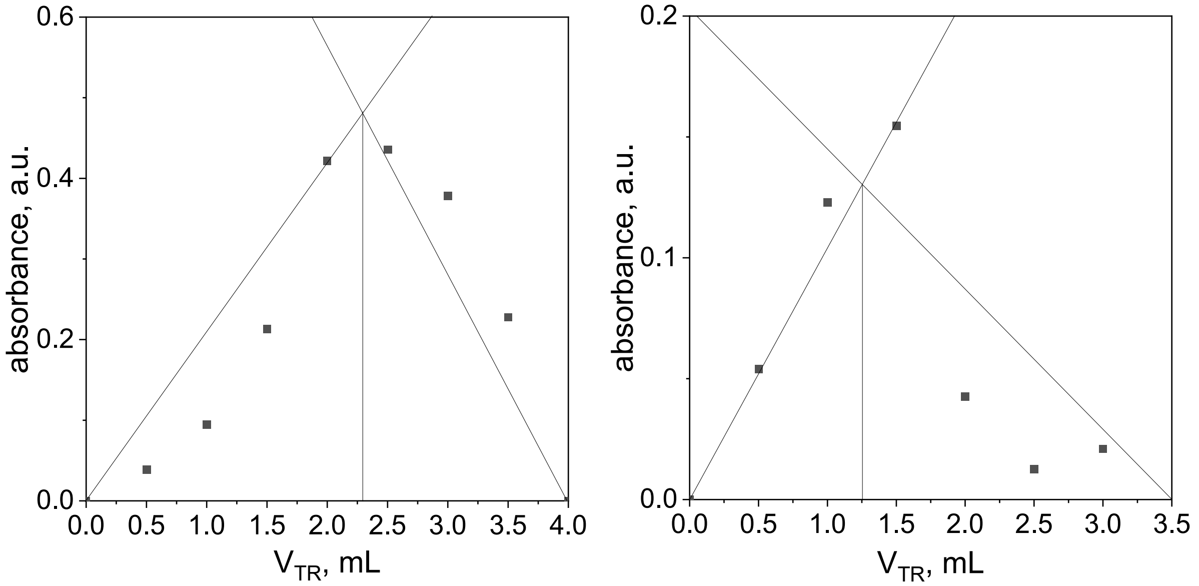

(**a**) (**b**)

Figure S21. The dependency of absorbance vs. ratio of tropaeolin to Pd(II) volumes, registered at 559 nm (b); and at 703 nm (c) past 7 days. Conditions: $C_{0,TR} = C_{0,Pd(II)} = 5 \cdot 10^{-5}$ mol/dm^3, total volume of reagent = 4.0 mL, T = 20°C, pH = 4.10.

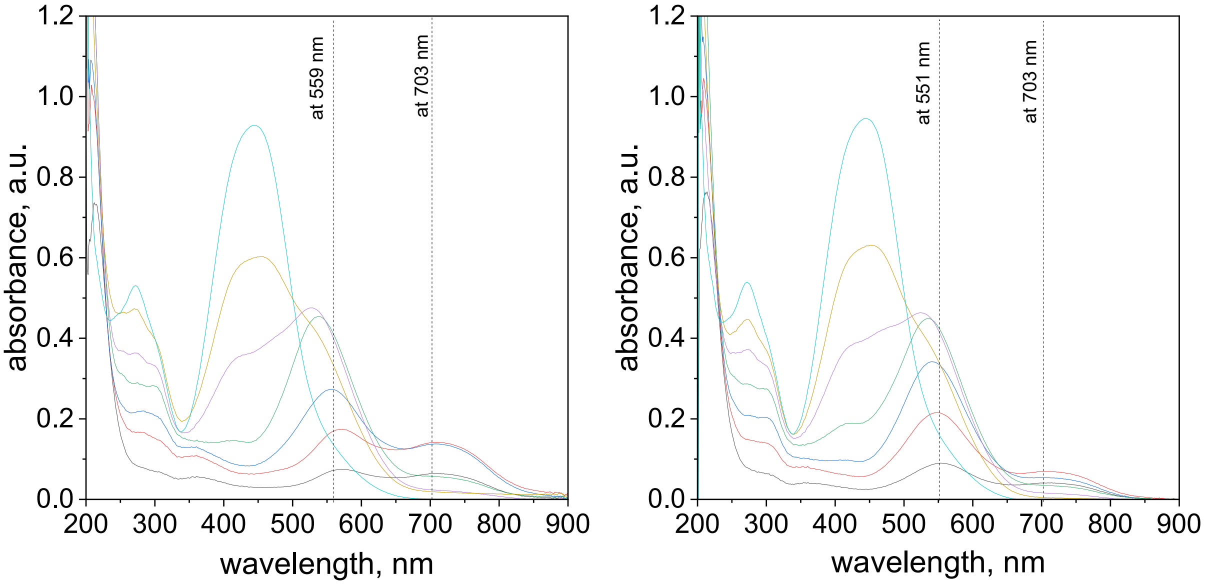

(**a**) (**b**)

Figure S22. The UV-Vis spectra of solutions obtained after mixing of Pd(II) ions with tropaeolin at different volumetric ratio ($V_{Pd(II)} : V_{TR}$) 3.5 : 0.5 (A); 3.0 : 1.0 (B); 2.5 : 1.5 (C); 2.0 : 2.0 (D), 1.5 : 2.5 (E); 1.0 : 3.0 (F), 0.5 : 3.5 (G), past 24h (a) and 7 days (b). Conditions: $C_{0,TR} = C_{0,Pd(II)} = 5 \cdot 10^{-5}$ mol/dm^3 (before reagent mixing), T = 20°C, pH = 5.02.

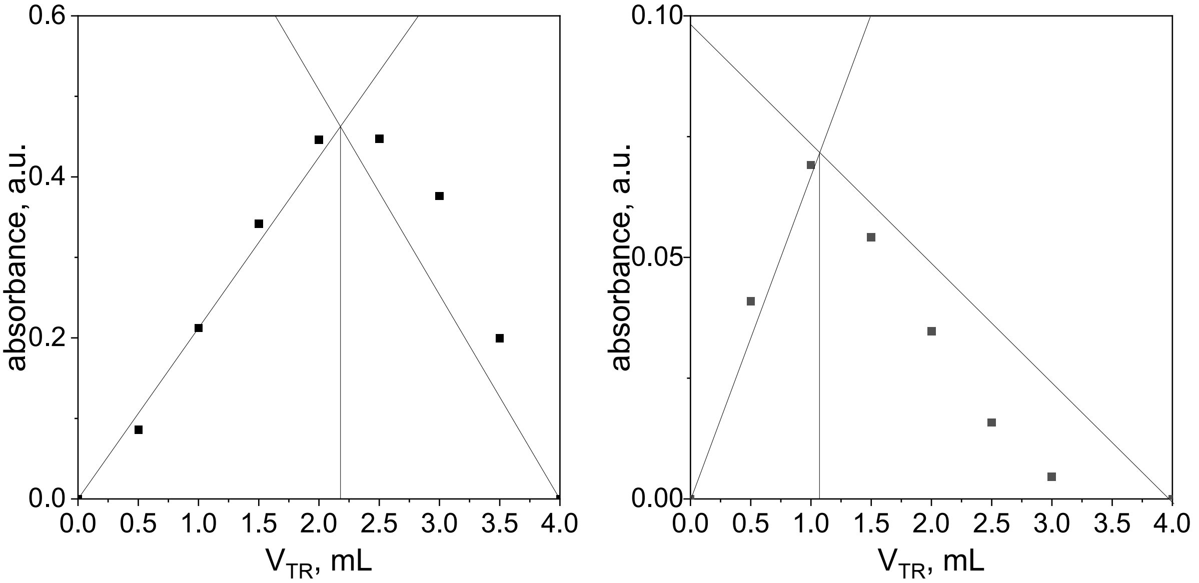

(**a**) (**b**)

Figure S23. The dependency of absorbance vs. ratio of tropaeolin to Pd(II) volumes, registered at 551 nm (b); and at 703 nm (c) past 24h. Conditions: $C_{0,TR} = C_{0,Pd(II)} = 5 \cdot 10^{-5}$ mol/dm^3, total volume of reagent = 4.0 mL, T = 20°C, pH = 5.02.

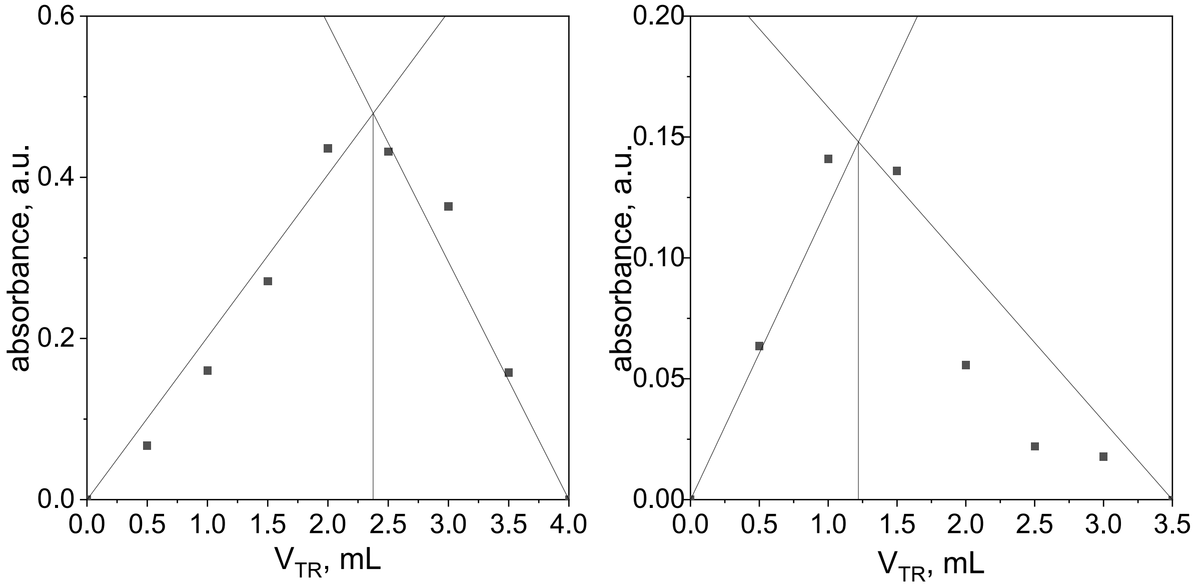

(**a**) (**b**)

Figure S24. The dependency of absorbance vs. ratio of tropaeolin to Pd(II) volumes, registered at 551 nm (b); and at 703 nm (c) past 7 days. Conditions: $C_{0,TR} = C_{0,Pd(II)} = 5 \cdot 10^{-5}$ mol/dm^3, total volume of reagent = 4.0 mL, T = 20°C, pH = 5.02.

4. DFT calculations for Tropaeolin OO

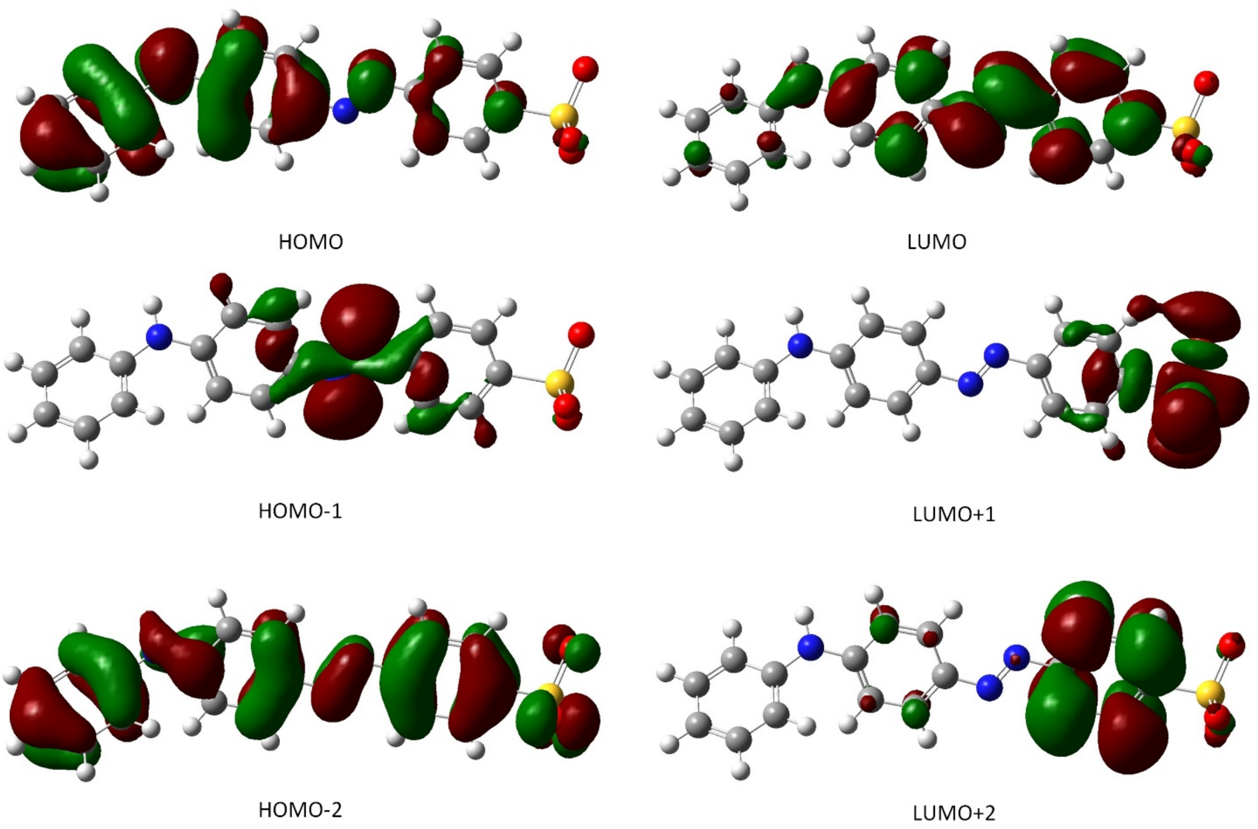

Figure S25. The HOMO and LUMO for tropaeolin OO.

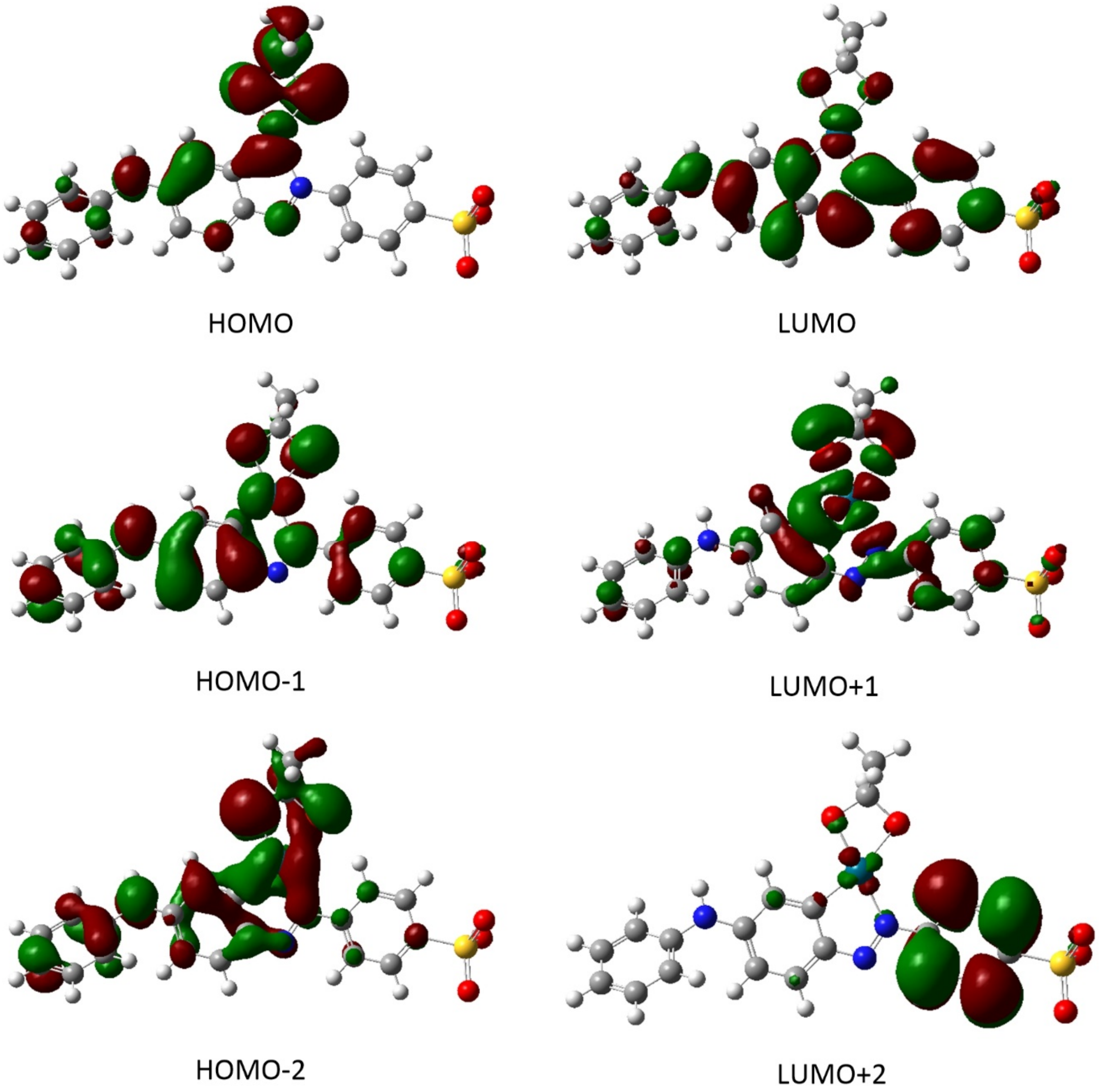

Figure S26. The HOMO and LUMO for complex TR-Pd 1

5. The influence of temperature

Table S2. The change of wavelength at which the maximum on UV–Vis spectrum was observed. Conditions: the value of concentration of Pd(II) ions after mixing with tropaeolin: $C_{0,MO}$ = 1.875· 10^{-5} mol/dm^3, $C_{0,Pd(II)}$ = 3.125· 10^{-5} mol/dm^3, T = 20–60°C, pH = 4.1. Spectra recorded after 17.5 hours.

Temperature [°C]	λ_1 nm	λ_2 nm	λ_3 nm	λ_4 nm
20	277	355	544	703
30	280	355	550	703
40	280	355	553	703
50	280	359	560	703
60	280	358	573	703

6. The influence of chloride ions on process of Pd(II) ions determination

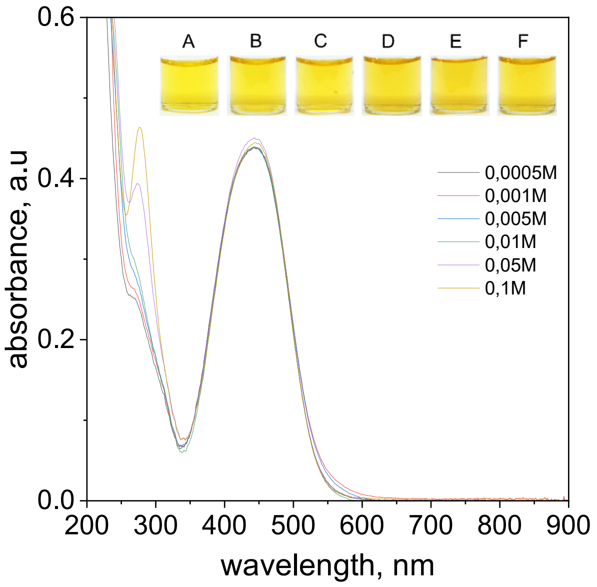

Figure S27. The UV-Vis spectra coming from solution contains the mixture of tropaeolin with Pd(II) and different concentrations of Cl^- (0,1M - 0,0005M), after 5 min. Conditions: volumetric ratio: 2,5mL Pd(II) : 1,5mL TR, the value of concentration of reagents after mixing: TR= 1,875·10^{-5} mol/dm^3, Pd(II)= 3,125·10^{-5} mol/dm^3, pH = 4.10, temperature 50°C.

7. The influence of chlorate ions on process of Pd(II) ions determination

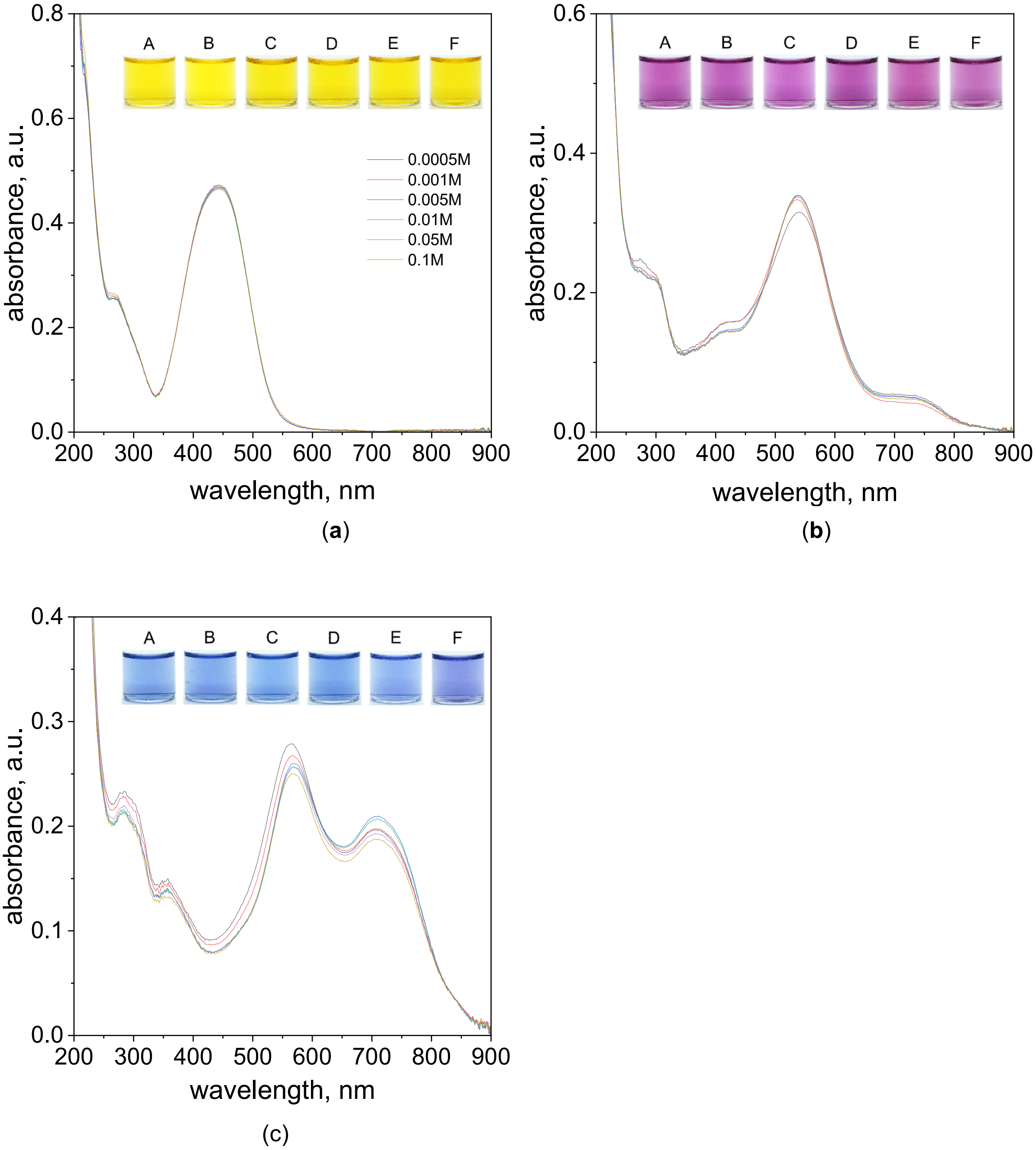

Figure S28. The UV-Vis spectra coming from solution contains the mixture of tropaeolin with Pd(II) and different concentrations of ClO_4^- A: 0.1 mol/dm^3, B: 0.05 mol/dm^3, C: 0.01 mol/dm^3, D: 0.005 mol/dm^3, E: 0.001 mol/dm^3, F: 0.0005 mol/dm^3 after (a) 5 min, 1 h, (b) 24 h. Conditions: volumetric ratio: 2.5 mL Pd(II) : 1.5mL TR, the value of concentration of Pd(II) ions after mixing with tropaeolin TR = 1.875·10^{-5} mol/dm^3, Pd(II) = 3.125·10^{-5} mol/dm^3, pH = 4.10, temperature 50°C.

8. The influence of presence other cations on process of Pd(II) ions determination

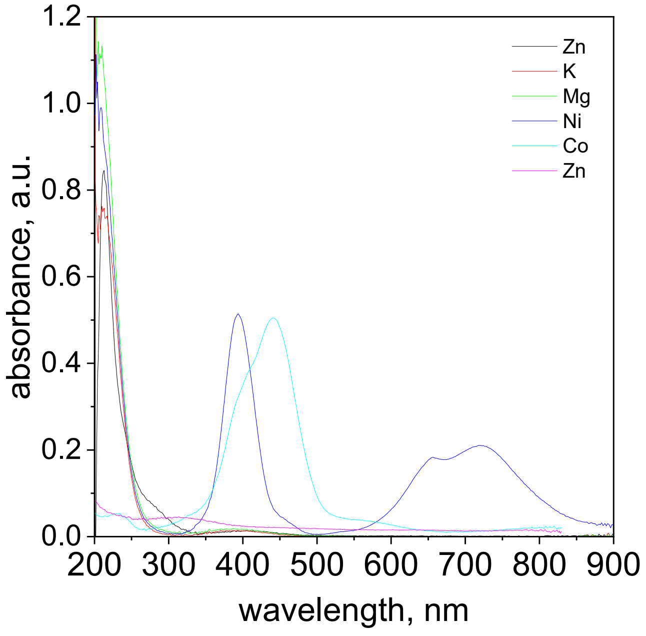

(**a**)

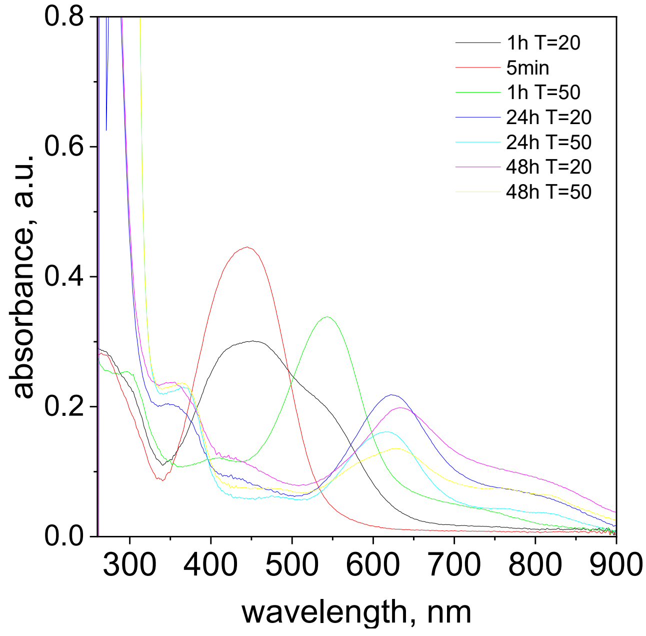

(**b**)

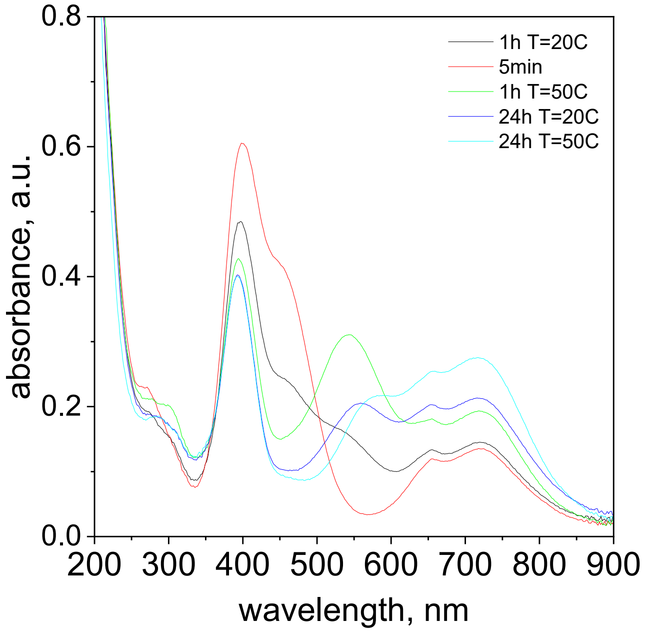

(c)

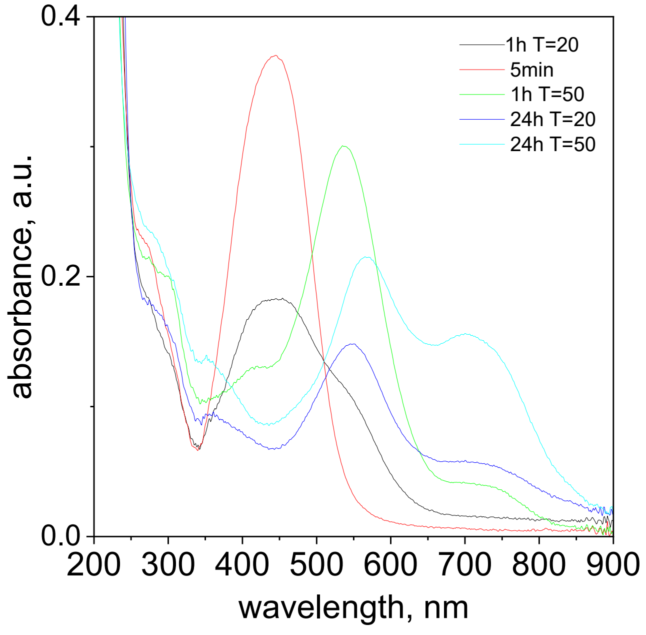

(d)

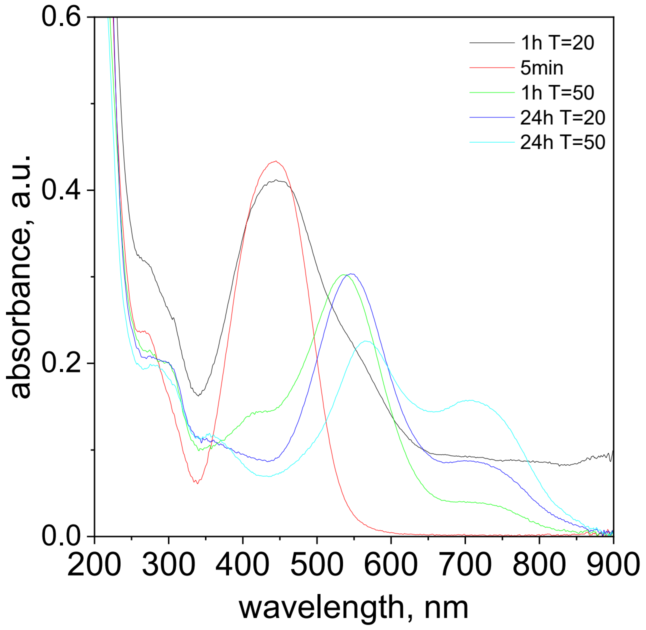

(e)

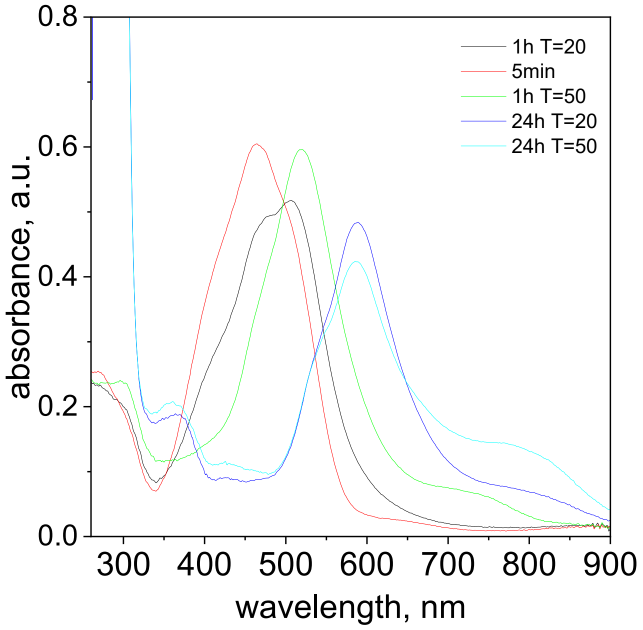

(f)

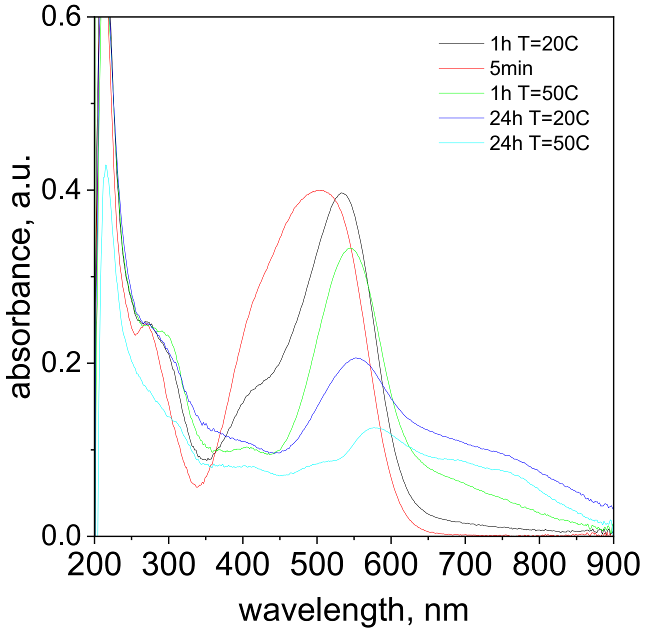

(g)

Figure S29. The evolution of UV - Vis spectra solutions containing Pd(II) and other metal cations (a), mixing Pd(II), tropaeolin and other metal cations: Zn^{2+} (b), Ni^{2+}(c), Mg^{2+} (d), K^{+} (e), Co^{2+} (f), Al^{3+}(g), after 5 min, 1 h and 24 h in temperature 20°C and 50°C. Conditions: $C_{0,TR}$ = 1,875·10^{-5} mol/dm^3, $C_{0,Pd(II)=}$ 3,125·10^{-5} mol/dm^3, $C_{0,anions}$ = 0.0625 mol/dm^3, volumetric ratio mixing Pd(II) ions and tropaeolin 2.5mL: 1.5mL, pH = 4.10.

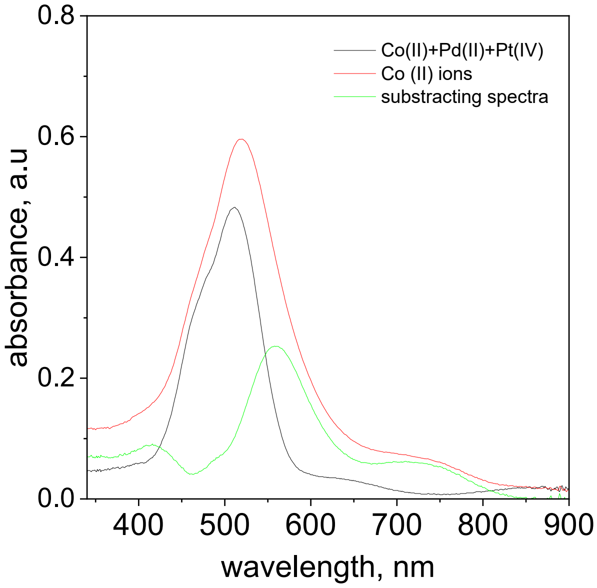

Figure S30. The evolution of UV - Vis spectra solutions containing TR, Pd(II), Pt(IV) and Co(II), single Co(II) ion and subtracting both spectra, after 1 h in temperature 50°C. Conditions: $C_{0,TR}$ = 1,875·10^{-5} mol/dm^3, $C_{0,Pd(II)}$= 3,125·10^{-5} mol/dm^3, $C_{0,Co}$ = 0.0625 mol/dm^3, volumetric ratio mixing Pd(II) ions and tropaeolin 2.5mL: 1.5mL, pH = 4.10.

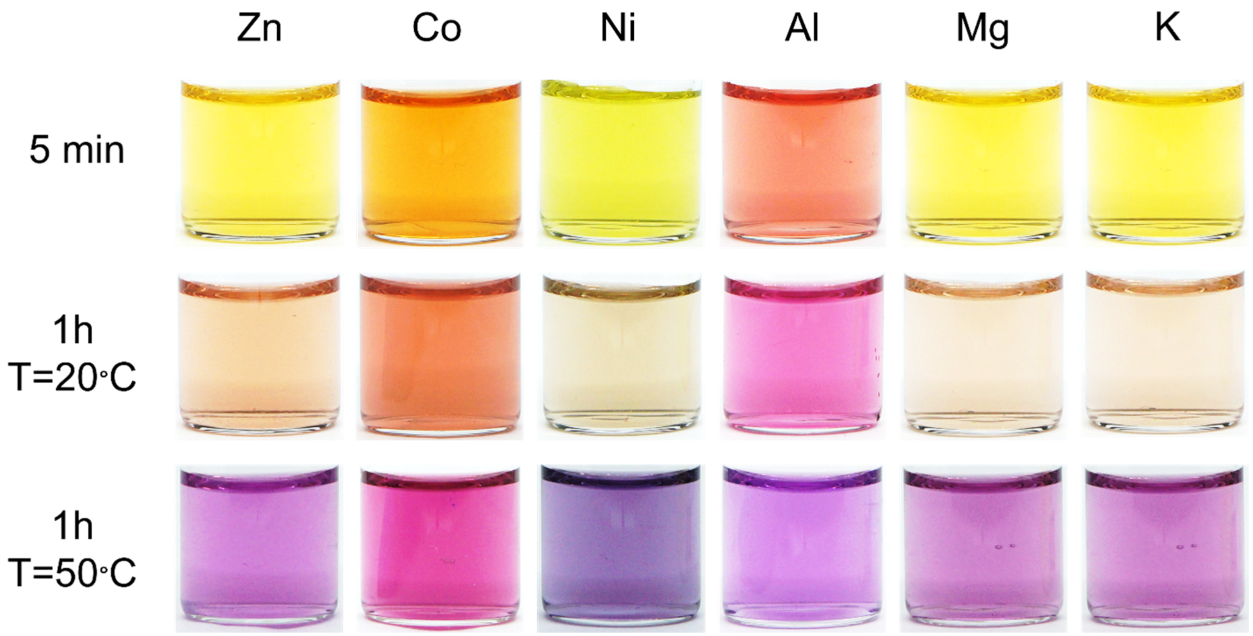

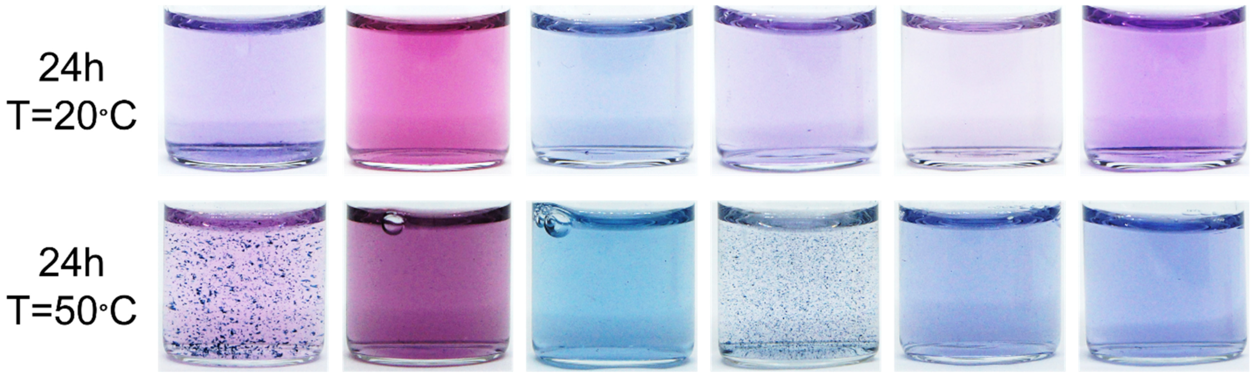

Figure S31. The colour change of mixture solutions containing Pd(II), tropaeolin and other metal cations: Zn^{2+}, Ni^{2+}, Mg^{2+}, K^{+}, Co^{2+}, Al^{3+}, after 5min, 1h and 24h in temperature 20°C and 50°C. Conditions: $C_{0,TR}$ = 1,875·10^-5 mol/dm^3, $C_{0,Pd(II)}$= 3,125·10^-5 mol/dm^3, $C_{0,anions}$ = 0.0625 mol/dm^3, volumetric ratio mixing Pd(II) ions and tropaeolin 2.5mL: 1.5mL, pH = 4.10.

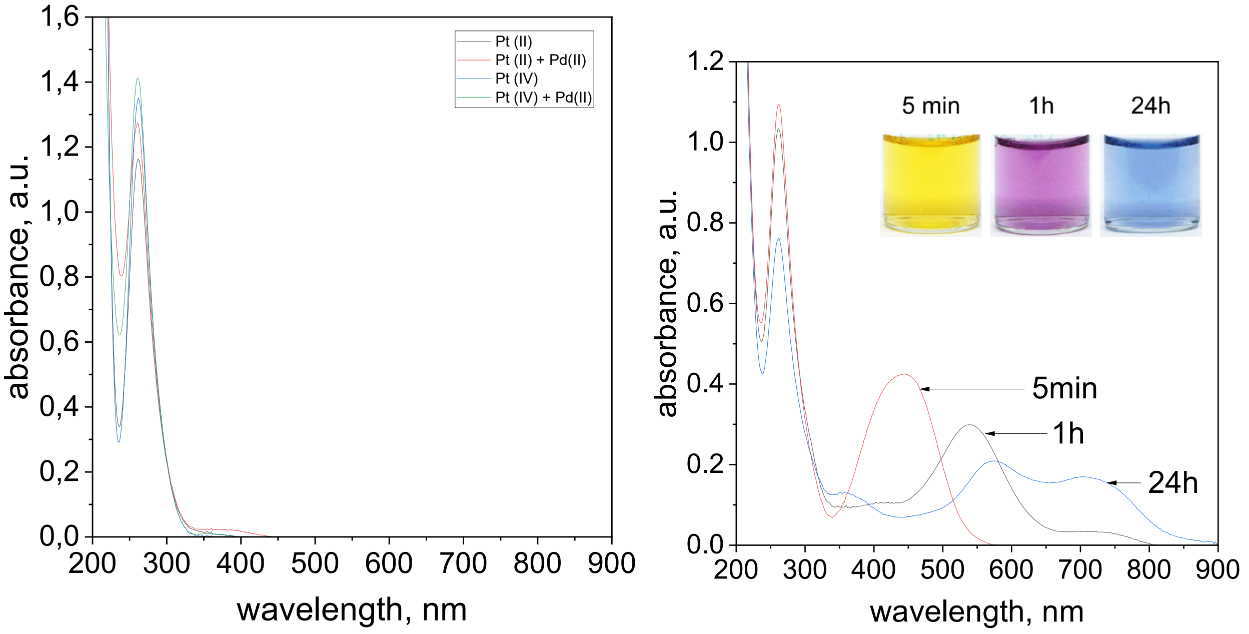

(a) (b)

Figure S32. The UV-Vis spectra coming from solution contains single Pt(II), Pt(IV), mixing Pd(II) + Pt(II) and Pd(II) + Pt(IV) (a). Mixture of tropaeolin with Pt(IV) + Pd(II) after 5 min, 1 h and 24 h (b). Conditions: $C_{0,TR}$ = 1.875·10^-5 mol/dm^3, $C_{0,Pt(II)/Pt(IV)}$= 3.125·10^-5 mol/dm^3, volumetric ratio mixing Pd(II) ions and tropaeolin 2.5mL: 1.5mL, pH = 4.10, T = 50°C .

9. Applying proposed method in the practice for spectrophotometric determination of Pd(II) ions.

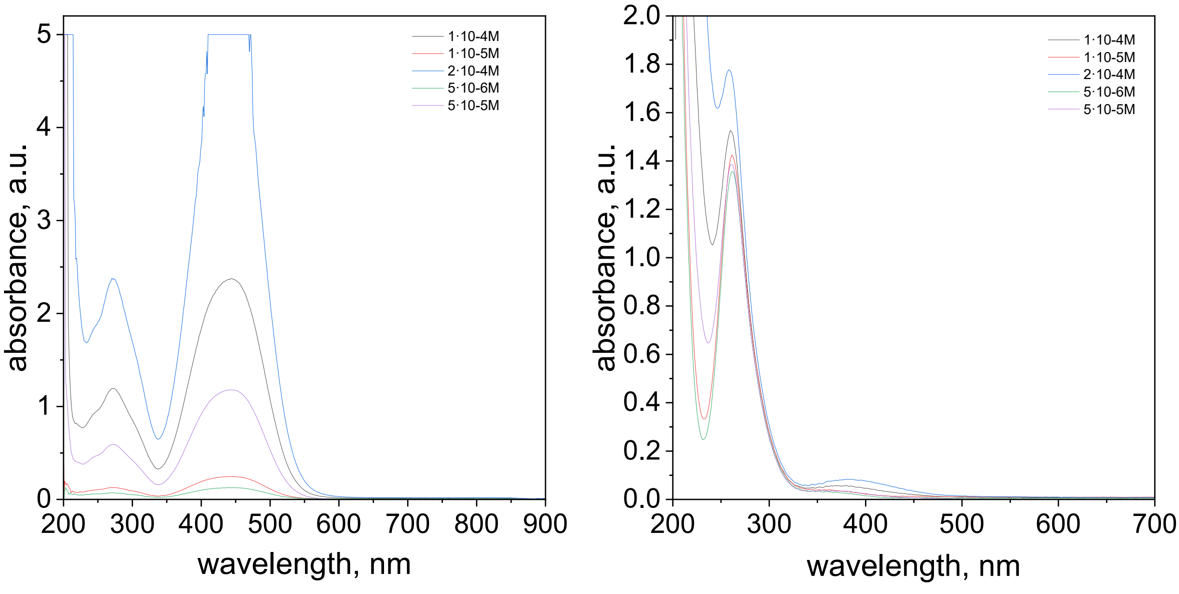

(**a**) (**b**)

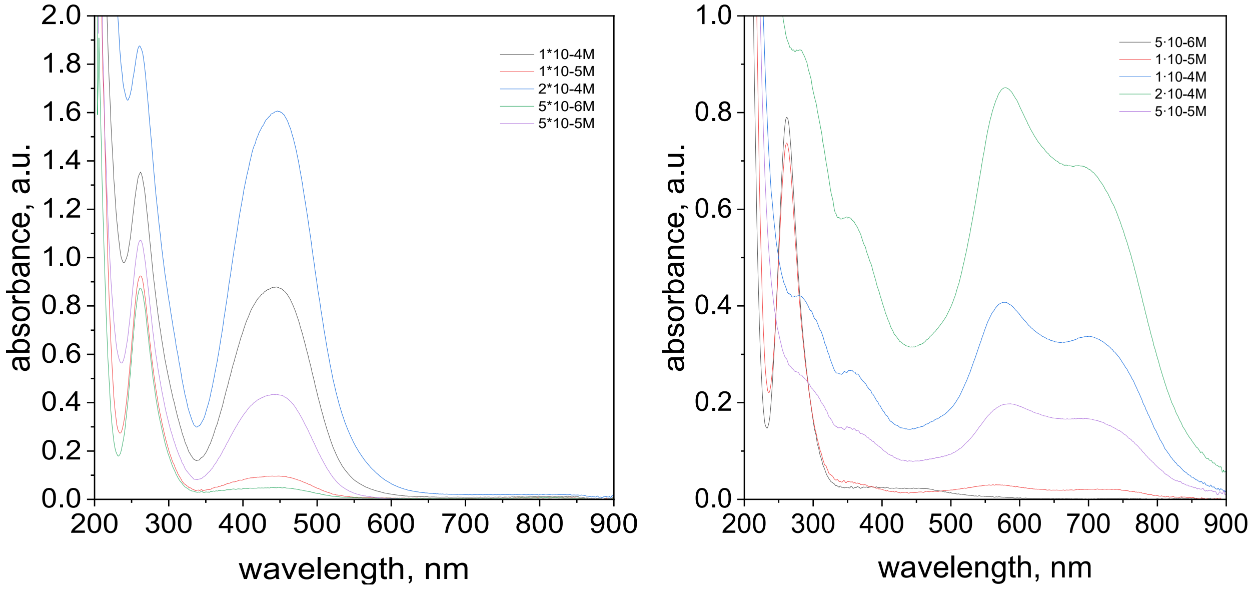

(c) (d)

Figure S33. The UV-Vis spectra coming from solution contains tropaeolin in concentration range from $5 \cdot 10^{-6}$ M to $2 \cdot 10^{-4}$ M (a), mixture of Pd(II) Pt(IV) in concentration range from $5 \cdot 10^{-6}$ mol/dm³ to $2 \cdot 10^{-4}$ (b), mixing of Pd(II), Pt(IV), TR after 5 min; (c) 24h (d). Conditions: $C_{0\ TR/C0Pd(II)}$ = $5 \cdot 10^{-6}$ mol/dm³, $5 \cdot 10^{-5}$ mol/dm³, $1 \cdot 10^{-5}$ mol/dm³, $1 \cdot 10^{-4}$ M, $2 \cdot 10^{-4}$ mol/dm³, $C_{0Pt(IV)}$ = $5 \cdot 10^{-5}$ mol/dm³, volumetric ratio mixing Pd (II) ions and tropaeolin 2.5mL: 1.5mL, pH = 4.10, T=60°C.

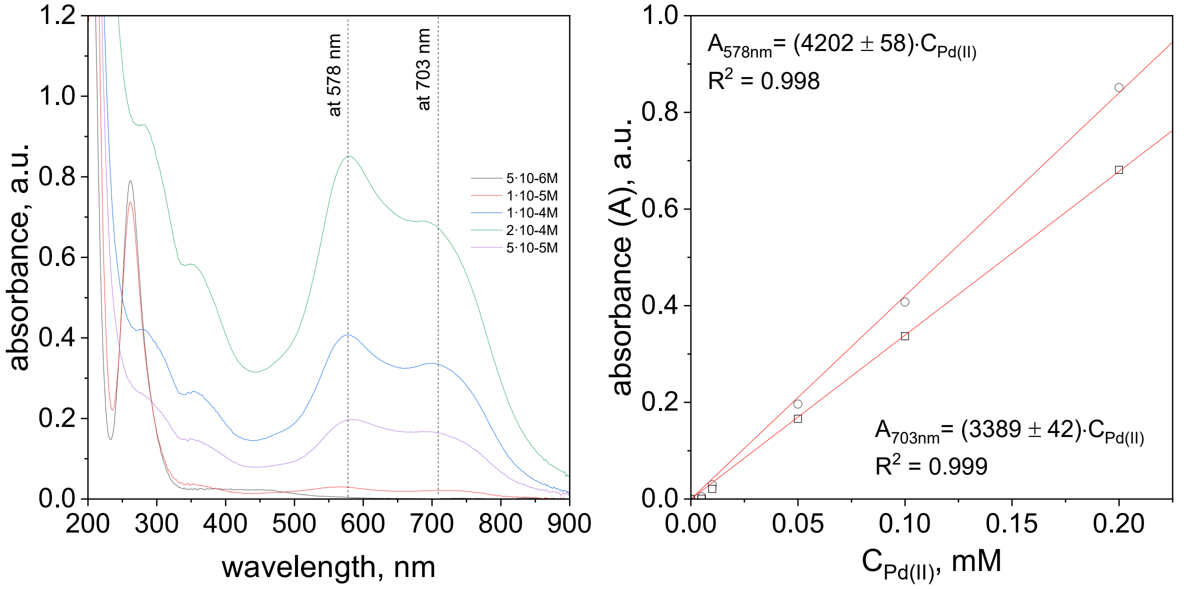

(**a**) (**b**)

Figure S34. The UV-Vis spectra coming from solution contains mixing of Pd(II), Pt(IV), TR after 24 h (a), dependency of absorbance vs different concentrations of Pd (II) ions after 24 h, in wavelength: 578 nm and 708 nm (b). Conditions: $C_{0\ TR/C0Pd(II)}$ = $5 \cdot 10^{-6}$ mol/dm³, $5 \cdot 10^{-5}$ mol/dm³, $1 \cdot 10^{-5}$ mol/dm³, $1 \cdot 10^{-4}$ mol/dm³, $2 \cdot 10^{-4}$

mol/dm^3, $C_{0Pt(IV)}$ = 5·10^{-5} mol/dm^3, volumetric ratio mixing Pd (II) ions and tropaeolin 2.5mL: 1.5mL, pH = 4.10, T= 60°C.

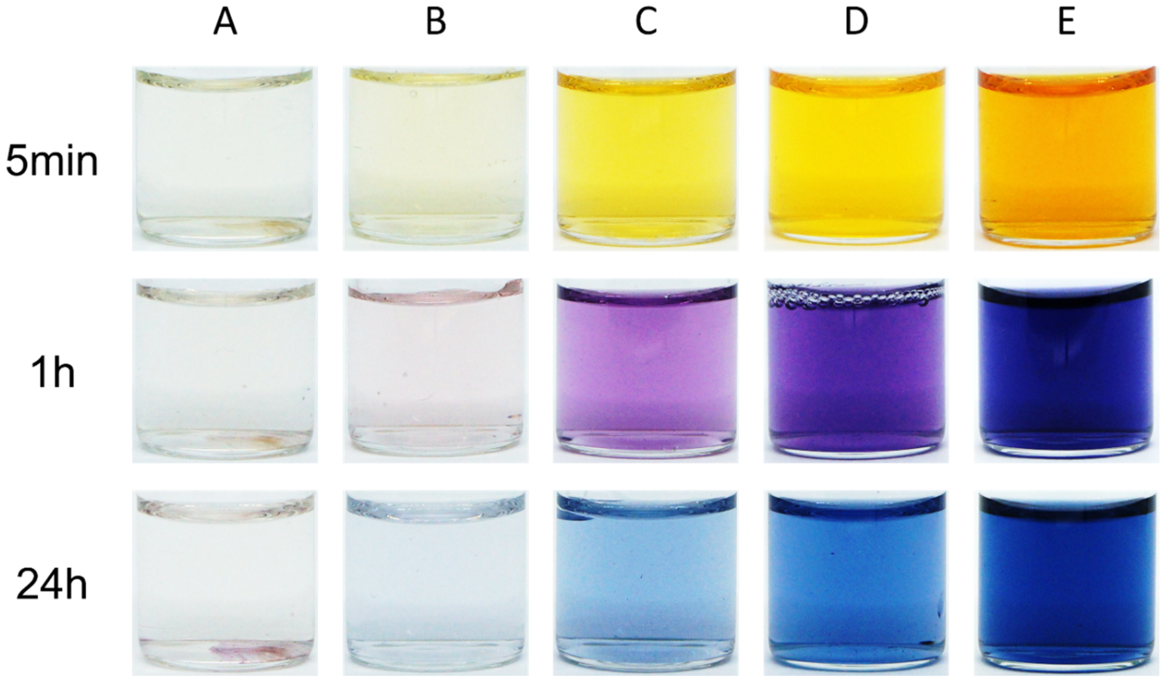

Figure S35. The colour change of mixture solutions containing TR, Pd(II), Pt(IV) ions, after (a) 5min; (b) 1h; (c) 24h, with different concentrations of Pd(II) and TR: A – 5·10^{-6} mol/dm^3,; B – 5·10^{-5} M; C –1·10^{-5} M M; D – 1·10^{-4} M, M; E–2·10^{-4} M. Conditions: $C_{0\ TR/C0Pd(II)}$ = 5·10^{-6} mol/dm^3, 5·10^{-5} mol/dm^3, 1·10^{-5} mol/dm^3, 1·10^{-4} mol/dm^3, 2·10^{-4} mol/dm^3, $C_{0Pt(IV)}$ = 5·10^{-5} mol/dm^3, volumetric ratio mixing Pd(II) ions and tropaeolin OO 2.5mL: 1.5mL, pH = 4.10, T=60°C
